# Plasma-borne indicators of inflammasome activity in Parkinson’s disease patients

**DOI:** 10.1038/s41531-020-00147-6

**Published:** 2021-01-04

**Authors:** Faith L. Anderson, Katharine M. von Herrmann, Angeline S. Andrew, Yuliya I. Kuras, Alison L. Young, Clemens R. Scherzer, William F. Hickey, Stephen L. Lee, Matthew C. Havrda

**Affiliations:** 1 Department of Molecular and Systems Biology, Geisel School of Medicine at Dartmouth College, Hanover, NH USA; 2 Department of Community and Family Medicine, Geisel School of Medicine at Dartmouth College, Hanover, NH USA; 3 APDA Center for Advanced Parkinson Research, Harvard Medical School, Brigham & Women’s Hospital, Boston, MA USA; 4 Department of Pathology, Dartmouth-Hitchcock Medical Center, Lebanon, NH USA; 5 Department of Neurology, Dartmouth-Hitchcock Medical Center, Lebanon, NH USA

**Keywords:** Parkinson's disease, Inflammation, Biomarkers

## Abstract

Parkinson’s disease (PD) is a neurodegenerative disorder characterized by motor and non-motor symptoms and loss of dopaminergic neurons of the substantia nigra. Inflammation and cell death are recognized aspects of PD suggesting that strategies to monitor and modify these processes may improve the management of the disease. Inflammasomes are pro-inflammatory intracellular pattern recognition complexes that couple these processes. The NLRP3 inflammasome responds to sterile triggers to initiate pro-inflammatory processes characterized by maturation of inflammatory cytokines, cytoplasmic membrane pore formation, vesicular shedding, and if unresolved, pyroptotic cell death. Histologic analysis of tissues from PD patients and individuals with nigral cell loss but no diagnosis of PD identified elevated expression of inflammasome-related proteins and activation-related “speck” formation in degenerating mesencephalic tissues compared with controls. Based on previous reports of circulating inflammasome proteins in patients suffering from heritable syndromes caused by hyper-activation of the NLRP3 inflammasome, we evaluated PD patient plasma for evidence of inflammasome activity. Multiple circulating inflammasome proteins were detected almost exclusively in extracellular vesicles indicative of ongoing inflammasome activation and pyroptosis. Analysis of plasma obtained from a multi-center cohort identified elevated plasma-borne NLRP3 associated with PD status. Our findings are consistent with others indicating inflammasome activity in neurodegenerative disorders. Findings suggest mesencephalic inflammasome protein expression as a histopathologic marker of early-stage nigral degeneration and suggest plasma-borne inflammasome-related proteins as a potentially useful class of biomarkers for patient stratification and the detection and monitoring of inflammation in PD.

## Introduction

Parkinson’s disease (PD) is a complex age-related neurodegenerative disease impacting multiple systems and characterized clinically by a host of motor and non-motor symptomologies^1,2^. Diagnostic tests and neuroprotective strategies have been slow to emerge in part because the molecular mechanisms of disease initiation and progression are not completely defined^3,4^. Neuroinflammation and mesencephalic dopaminergic neuronal cell loss are observable in post-mortem tissues obtained from PD patients^5^ and it is widely believed that inflammation contributes to the progression of the neurodegenerative process^6,7^. Evidence supporting a role for neuroinflammation in PD comes from animal models^8,9^ and epidemiologic studies indicating that the use of non-steroidal anti-inflammatory drugs (NSAIDs), specifically ibuprofen, are associated with a reduced risk of developing PD^10,11^. It has been estimated that 30–70%^12,13^ of dopaminergic neurons are already lost at the time of motor symptom onset and diagnosis of PD. It is imperative that we leverage our growing understanding of PD-associated pathophysiologies like inflammation to help us understand the complexity of PD and inform the development of preventative measures and neuroprotective strategies to avoid onset or slow the progression of the disease.

Inflammasomes are pro-inflammatory intracellular complexes defined by proteins belonging to the nucleotide-binding domain-like receptors (NLRs), absent in melanoma 2-like receptors (ALRs), or pyrin pattern recognition receptor families^14^. The assembly of the inflammasome complex drives caspase-1 catalysis to initiate cytokine maturation and secretion, and potentially pyroptosis, an inflammatory subcategory of programmed cell death^15,16^. Inflammasomes are increasingly linked to neurodegenerative diseases, especially Alzheimer’s disease^17,18^ and PD^19^. Such associations are intuitive because NLRP3 inflammasome activity can be triggered by sterile cellular stress associated with neurodegenerative disease pathology including cell death^20,21^, mitochondrial stress^22^, reactive oxygen species (ROS)^23^, and proteinaceous insults including amyloid-β^24,25^ and α-synuclein^26^ aggregation. A growing body of evidence linking the NLRP3 inflammasome with PD includes recent reports characterizing a *Nlrp3* inflammasome response to fibrillar α-synuclein exposure^26,27^, ROS production^28^, and the occupational toxicant manganese^29^; three sterile, inflammatory triggers associated with the development and pathophysiology of PD^30^. Our studies have shown that: (1) the NLRP3 inflammasome is expressed in multiple cell types within the human mesencephalon^19^; (2) a polymorphism in *NLRP3* is associated with a reduced risk of PD^19^; and (3) that inactivation of *Nlrp3* is neuroprotective in a toxicant-based mouse model of PD^31^. Collectively, our findings and the reports of others suggest that inactivation of the NLRP3 inflammasome will likely benefit PD patients^27,32,33^.

A key aspect of inflammasome biology is the initiation of a subcategory of programmed cell death called pyroptosis. Unlike apoptosis, pyroptosis is pro-inflammatory and pyroptotic cells avidly release cytosolic proteins prior to cell death^34^. Canonical inflammasome signaling results in the release of cytokines including IL-1B, now known to often be released in membrane-bound vesicles^35^. Recent reports indicate that NLRP3 inflammasome activation can induce the cleavage of Gasdermin D (GSDMD)^36–38^. Once cleaved, GSDMD creates membrane pores inducing an osmotic shift that can result in cell rupture and the further release of intracellular proteins into the inflammatory environment^39^. The functional NLRP3 inflammasome includes an adapter protein called apoptosis associated speck-like protein containing a CARD (encoded by *PYCARD* or ASC herein). “ASC specks”^25^, as well as the NLRP3 protein itself^40^, have been observed in the extracellular space and can propagate inflammation in a paracrine manner^41^. The term “inflammasome-derived exosomes”^42^ was coined based on this realization that inflammasome activity results in the release of a pro-inflammatory class of extracellular vesicles (EVs) containing typically cytosolic proteins into the extracellular space. Our current understanding of inflammasomes in PD suggests that pyroptosis may be ongoing in response to a variety of sterile triggers associated with metabolic stress, proteinopathy, and cell death. We reason that inflammasome-related proteins may therefore be released and detectable in PD biofluids, serving as potentially useful indicators of sterile inflammation occurring in association with disease progression.

The NLRP3 inflammasome has emerged as both a key mediator of inflammation in the degenerating brain and a tractable therapeutic target^43,44^. Here, we characterized the NLRP3 inflammasome in PD patients with an emphasis on providing data to support the further development and implementation of methodologies for analysis of inflammasome activity during the progression of PD. Our patient-based study provides a characterization of NLRP3 in early stages of mesencephalic neurodegeneration and in biofluids obtained from PD patients. This study suggests that pyroptosis is a detectable feature of PD, and that inflammasome-associated proteins, including NLRP3, may represent a class of sorely needed peripheral biomarkers for disease-associated inflammation.

## Results

### Elevated expression of inflammasome-related proteins during multiple stages of nigral degeneration

**Fig. 1:**
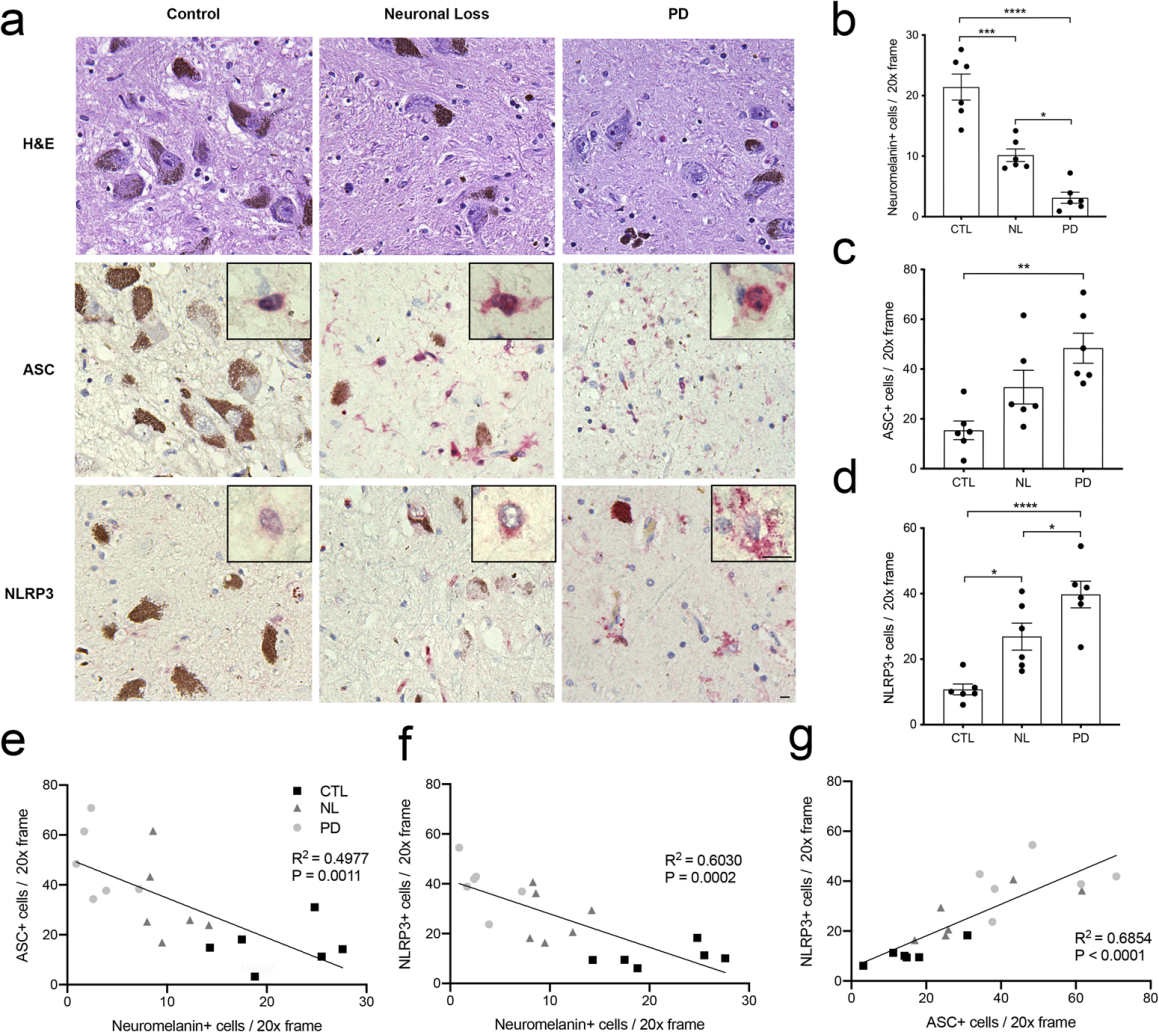
Elevated ASC and NLRP3 protein expression observed during multiple stages of mesencephalic neurodegeneration. **a** Post-mortem mesencephalic tissues were obtained from healthy control subjects (CTL) (*n* = 6), asymptomatic subjects with loss of SNpC neurons (NL) (*n* = 6), and clinically confirmed PD patients (PD) (*n* = 6). Tissues were sectioned at 5 µM and stained with H&E (top row), anti-ASC (middle row, red), or anti-NLRP3 antibodies (bottom row, red). Scale bars in 20× field and 60× inset represent 10 μm. **b** Fewer neuromelanin-positive neurons were observed in the SNpC in both NL tissues and PD tissues relative to healthy controls (****P < 0.0001, ***P = 0.0002, *P = 0.0111). **c** More ASC-positive cells were observed in the PD tissues relative to controls (**P = 0.0024). **d** More NLRP3-positive cells were observed in the NL tissues relative to CTL tissues (*P = 0.0132), while there were significantly more NLRP3-positive cells observed in the PD tissues relative to NL tissues (*P = 0.0487) and CTL tissues (****P < 0.0001). All quantification was performed by counting 10 distinct fields per section. *P*-values were determined using one-way ANOVA with multiple comparisons, error bars in **b**–**d** represent s.e.m. **e** ASC-immunoreactive cells compared to neuromelanin-positive cells per tissue in three histologic groups (*R*^2^ = 0.4977, *P* = 0.0011). **f** NLRP3-immunoreactive cells were statistically associated with reduced number of neuromelanin-positive cells across three histologic groups (*R*^2^ = 0.6030, *P* = 0.0002). **g** Correlation between number of ASC-immunoreactive and NLRP3-immunoreactive cells (*R*^2^ = 0.6854, *P* < 0.0001).

We and others report enhanced expression of inflammasome-related proteins in brain tissues obtained post-mortem from patients with late-stage PD^19,27^. To determine if elevation of inflammasome-related protein expression is an early event in the degeneration of the mesencephalon, we interrogated 335 banked post-mortem tissues obtained between 2013 and 2017. We identified 24 individuals whose pathological reports cited evidence of mesencephalic neuronal cell loss but who were neither diagnosed with PD nor displayed clinical symptomology. We analyzed a subset of these individuals in comparison with tissues obtained from healthy controls and late stage, histopathologically confirmed clinically diagnosed PD patients (Supplemental Table 1). Mesencephalic tissues were stained with H&E (top row), anti-ASC antibodies (middle row), or anti-NLRP3 antibodies (bottom row) (Fig. 1a). To verify pathology reports, we quantified the number of pigmented neurons in the substantia nigra pars compacta (SNpC) based on the easily recognizable presence of intracellular neuromelanin granules (Fig. 1a, top row, brown). Consistent with the initial pathologic determination, there were fewer pigmented neurons in the SNpC of the patients observed to have nigral cell loss compared with healthy controls (Fig. 1b). The number of pigmented neurons in the nigral cell loss patients was intermediate compared with healthy controls and confirmed PD cases (Fig. 1b) indicating that these tissues were reasonable surrogates for early-stage degeneration of the SNpC. Microscopic examination of these tissues revealed an easily observable increase in the intensity of both ASC and NLRP3 immunoreactivity in PD cases compared with controls and again indicated an intermediate phenotype in nigral cell loss patients (Fig. 1a, middle and bottom rows). There was an increase in number of ASC and NLRP3-immunoreactive cells in PD cases compared with controls and an intermediate phenotype in nigral cell loss patients (Fig. 1c, d). The number of ASC and NLRP3-immunoreactive cells correlated with nigral cell loss (Fig. 1e, f) and ASC and NLRP3-immunoreactive cells were positively correlated with one another (Fig. 1g). To determine cell-type specificity, we performed immunofluorescent co-localization studies using confocal microscopy. Sections of a representative SNpC were co-stained with anti-NLRP3 antibodies along with either anti-IBA1, anti-GFAP, or anti-TH antibodies to highlight microglia, astrocytes, and dopaminergic neurons, respectively. Consistent with previous reports^24,45^, we found NLRP3 co-localized with IBA1 (Supplemental Fig. 1). NLRP3 specks were in close proximity to GFAP staining in these tissues (Supplemental Fig. 1). We also observed NLRP3 and TH colocalization (Supplemental Fig. 1), an observation that we previously reported in patient tissues^19^ and have evaluated in detail using a newly developed mouse model^46^. These studies confirm previous reports from our laboratory^19^ and others^27^ indicating enhanced inflammasome-related protein expression in the degenerating mesencephalon in PD, and extend these studies by characterizing the expression of inflammasome-related proteins in the degenerating SNpC of individuals with nigral cell loss not identified to have clinical symptoms of PD.

### Characterizing potential origins of inflammasome activity in PD

Detecting chronic inflammation in living patients with PD is challenging because methods to non-invasively measure slowly progressive changes are limited. While certain cytokines are elevated in PD biofluids, such as IL-6, IL-2, IL-8, IL-10, MCP-1, RANTES, MIP-1α, IFNγ, IL-1B, and TNFα^47–49^, none have gained traction as clinical diagnostic indicators. We reasoned that sterile pyroptotic processes such as those driven by NLRP3 may result in the release of previously uncharacterized cytosolic proteins, expanding the pool for discovery of useful molecules for monitoring inflammation in PD. Previous studies of microglia support a model in which pyroptosis-related proteins are released from activated central nervous system cells^29^. Consistent with these reports^45,50^, when treated sequentially with the well-characterized NLRP3 inflammasome activators lipopolysaccharide (LPS) and nigericin, we detected multiple cytosolic pyroptosis-related proteins in the conditioned media of microglia-enriched primary glial cultures obtained from wild-type (WT) and *Nlrp3*^*−/*−^ mice (Supplemental Fig. 2a). SDS–PAGE studies readily confirmed the presence of extracellular, *Nlrp3*-dependent, NLRP3, ASC, CASP1, GSDMD, and the pro-form of IL-1B (Supplemental Fig. 2a, b). Indicative of inflammasome activation, we also identified the activated p10 fragment of CASP1 and the mature form of IL-1B in a *Nlrp3*-dependent manner (Supplemental Fig. 2a lower MW bands, and 2b). Further confirming the pyroptotic process, in the same conditioned media we observed *Nlrp3*-dependent cleaved GSDMD, a key marker distinguishing caspase 1-dependent pyroptosis from caspase 3-dependent apoptosis^16^ (Supplemental Fig. 2a, lower MW bands, and 2b). To more thoroughly characterize intracranial sources of pyroptotic activity, we evaluated a publicly available single cell transcriptomic dataset obtained from aged mice^51^. We confirmed that microglia were indeed a major cell type expressing the complete host of NLRP3 inflammasome components including *Nlrp3*, *Pycard*, *Casp1*, as well as direct targets and elicitors of inflammatory pyroptosis, *Gsdmd* and *IL-1b*, in the aged mouse brain (Supplemental Fig. 2c). Further inspection of the data presented in Supplemental Fig. 2c indicated other potential origins of inflammasome activity in the aged mouse brain including dendritic cells (DCs) and endothelial cells (ECs). Notably, significant numbers of monocytes (MNC) and macrophages (MAC) present in aged mouse brain tissues expressed the core components of the NLRP3 inflammasome. In light of these findings, we confirmed previous reports^52,53^ characterizing the release of pyroptosis-related proteins from THP-1 human monocytic cells stimulated with LPS and nigericin. As expected, we observed release of NLRP3, ASC, Pro-CASP1, and Pro-IL-1B, as well as the pyroptosis indicators p20 CASP1, cleaved GSDMD, and cleaved IL-1B following inflammasome activation as compared to vehicle-treated controls (Supplemental Fig. 3a, b). A lack of tractable methodologies for the characterization of cellular origins of circulating molecules in human studies is a limitation, however, consistent identification by our laboratory^31^ and others^27,45,52^ of extracellular inflammasome-related proteins being released by numerous cell types prompted us to evaluate the possibility that an inflammasome signature may be detectable in peripheral biofluids in PD patients.

### Elevated level of plasma-borne NLRP3 is associated with PD status

**Fig. 2:**
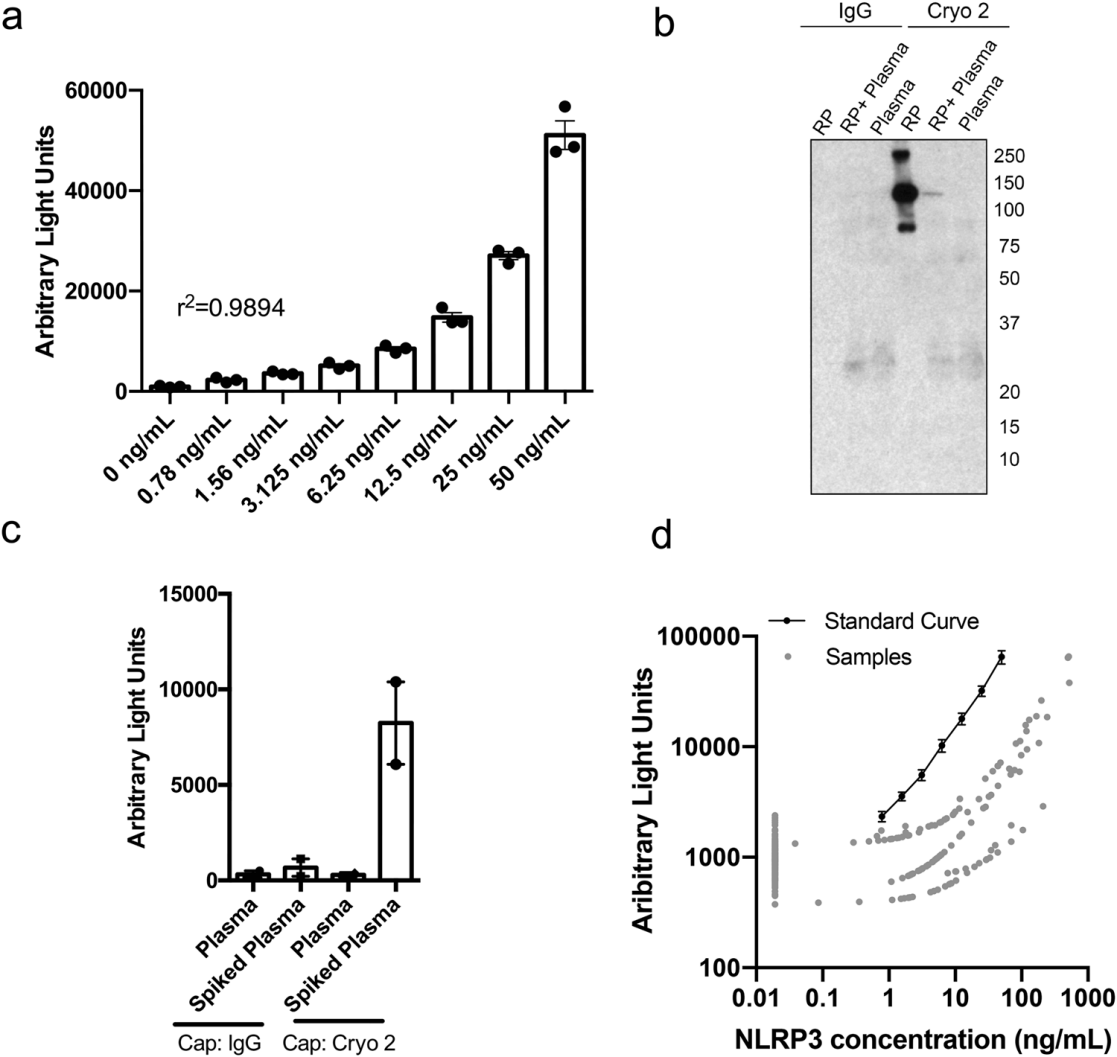
Development of an electrochemiluminescent-based assay for the detection of inflammasome-related protein NLRP3. Anti-NLRP3 antibodies were tested in different capture and detection antibody pairing orientations in 96-well electrode-containing immunosorbent plates (Meso Scale Diagnostics (MSD), Rockville, MD) for their ability to detect NLRP3 recombinant protein. Once an antibody pair was found, the capture antibody was biotinylated and assay conditions were optimized using streptavidin-coated 96-well electrode-containing immunosorbent plates. **a** The antibody pair identified was able to detect recombinant NLRP3 protein in 1% BSA (*r*^2^ = 0.9894) down to a concentration of 0.78 ng/mL. *R*^2^ was calculated using linear regression. **b** NLRP3 detection was confirmed through immunoprecipitation (IP), where the capture antibody was used as the pull-down antibody and the detection antibody was used as the immunoblotting antibody. **c** Clean plasma or plasma spiked with 12.5 ng/mL of recombinant NLRP3 protein was run on the optimized electrochemiluminescent assay using biotinylated mouse IgG or biotinylated Cryo 2 as the capture antibody in order to ensure antibody-pair specificity and low non-specific binding. **d** NLRP3 protein concentration was plotted against electrochemiluminescent read-out (Arbitrary Light Units) for the representative standard curve and all samples analyzed. Axes are on the logarithmic scale. Samples classified as “non-detectable” were imputed by taking the minimum positive value divided by 2. Error bars in **a** and **c** represent s.e.m.

Previous studies in cryopyrin-associated periodic syndrome (CAPS)^54^, myelodysplastic syndromes^55^, HIV^56^, and Sjögren’s syndrome^57^ support a model in which pyroptosis-related proteins are released and detectable in peripheral biofluids^29,58^. To detect plasma-borne pyroptosis-related proteins, we developed an electrochemiluminescence-based assay for the detection of NLRP3 in human plasma. Numerous commercially available antibodies for NLRP3 were analyzed in all possible combinations to identify candidate capture and detection pairs based on sensitivity and signal to noise ratio (Supplemental Table 2). Once an antibody pair was discovered, the assay was optimized using recombinant NLRP3 protein (RP) in 1% BSA determining a detection range of 50–0.78 ng/mL (Fig. 2a) and specificity was confirmed using an immunoprecipitation assay (Fig. 2b). Serial dilution assays were performed in a human plasma matrix to determine the optimal plasma dilution for detection of NLRP3 in human plasma with low background [1:16 (6.25%), Supplemental Fig. 4]. Assay performance and specificity was further verified by comparison with biotinylated mouse IgG, demonstrating minimal non-specific binding to isotype-matched control immunoglobulin (Fig. 2c). The parallelism of the standard curve and samples analyzed was confirmed, indicating that the matrix effect was minimal using the optimized conditions (Fig. 2d).

Analysis of plasma samples obtained from healthy controls and PD patients showed a modestly higher proportion of PD patients (18%) with elevated NLRP3 levels (in the 90th percentile), compared with healthy controls (10%) (Table 1, univariate *P*-value 0.086). Analysis of patient-completed surveys identified a higher proportion of PD patients reporting NSAID use (58%), compared with healthy controls (14%) (Table 1, univariate *P*-value < 0.001), and an association between plasma NLRP3 and use of certain NSAIDs and pain relievers (Table 2). When we excluded patients self-reporting use of NSAIDs at least weekly, the multivariable logistic regression analysis showed significant association between high levels of NLRP3 (>90th percentile) and PD (OR = 2.46, *P* = 0.033 adjusted for age and sex) (Table 3).

**Table 1:** Pooled population characteristics of samples obtained from Dartmouth Hitchcock Medical Center and the Harvard Biomarker Repository.

	Controls	PD	*P* univariate
	145	182	
Age (mean (SD))	69.89 (9.55)	70.03 (7.56)	0.88
Sex = Male (%)	62 (42.8)	105 (57.7)	0.01
Age of Onset (mean (SD))	n/a	62.78 (8.54)	n/a
Duration of disease (yrs) (mean (SD))	n/a	6.63 (4.97)	n/a
UPDRS Score (mean (SD))	n/a	38.69 (21.70)	n/a
DA replacement = Yes (%)	n/a	138 (78.9)	n/a
DA agonist = Yes (%)	n/a	66 (37.7)	n/a
DA anti-degradation = Yes (%)	n/a	76 (43.4)	n/a
NSAID = Yes (%)	20 (14.4)	98 (58.0)	<0.001
Acetaminophen^a^	4 (2.9)	20 (11.8)	0.007
Aspirin	6 (4.3)	25 (14.8)	0.004
Baby Aspirin	4 (2.9)	39 (23.1)	<0.001
Celebrex	0 (0.0)	1 (0.6)	1
Ibuprofen	8 (5.8)	33 (19.5)	0.001
Naproxen	0 (0.0)	20 (11.8)	<0.001
NLRP3 16.2+ ng/mL (%)—90th percentile	15 (10.3)	32 (17.7)	0.086

^a^Non-NSAID, non-aspirin pain reliever.

**Table 2:** Characteristics associated with elevated NLRP3.

	NLRP3 ng/mL		*P* univariate
	<16.2	16.2+	
	*N* = 279	*N* = 47	
Age (mean (SD))	70.33 (8.51)	67.91 (8.21)	0.071
Sex = Male (%)	140 (50.2)	27 (57.4)	0.45
NSAID = Yes (%)	101 (38.4)	16 (36.4)	0.93
Acetaminophen^a^	21 (8.0)	3 (6.8)	1
Aspirin	22 (8.4)	9 (20.5)	0.028
Baby Aspirin	38 (14.4)	5 (11.4)	0.76
Celebrex	1 (0.4)	0 (0.0)	1
Ibuprofen	39 (14.8)	2 (4.5)	0.11
Naproxen	15 (5.7)	4 (9.1)	0.60

^a^Non-NSAID, non-aspirin pain reliever.

**Table 3:** Elevated levels of NLRP3 are associated with PD once removing those with self reported NSAID use.

	Controls	PD	*P* univariate	Odds ratio^a^	(95% CI)	*P*^a^
	*N* = 119	*N* = 71				
Age (mean (SD))	69.95 (9.90)	68.42 (7.50)	0.26			
Sex = Male (%)	53 (44.5)	42 (59.2)	0.072			
NSAID = Yes (%)	None	None				
NLRP3 16.2+ ng/mL (%)—90th percentile	12 (10.1)	16 (22.5)	0.033	2.46	(1.08−5.76)	0.033

^a^Multivariable logistic regression model including age, sex (*P* = 0.033).

### Plasma-borne NLRP3 is indicative of inflammasome activation

**Fig. 3:**
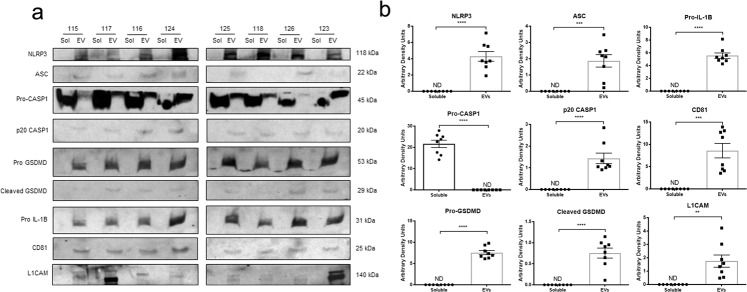
Inflammasome-related proteins are present in extracellular vesicles isolated from human plasma samples. **a** Extracellular vesicles (EVs) were isolated from fresh plasma samples using the ExoQuick Plasma Prep with Thrombin (EXOQ5TM-1, System Biosciences, Palo Alto, CA). The EV fractions were lysed and sonicated before analysis. The soluble and EV fractions from 8 plasma samples were analyzed via SDS–PAGE and subsequent immunoblotting. Anti-NLRP3, anti-ASC, anti-CASP1, anti-GSDMD, and anti-IL-1B antibodies were used for the detection of inflammasome-related proteins. Anti-CD81 and anti-L1CAM antibodies were used to confirm the purity of the EV fraction and assess the presence of neural-derived EVs, respectively. **b** The presence of inflammasome-related proteins was most often found solely in the EV fraction and the detection of EV-specific markers was found only in the EV fraction. All statistical analyses were performed using unpaired Students *t*-tests (NLRP3, ****P < 0.0001) (ASC, ***P = 0.0002) (Pro-CASP1, ****P < 0.0001) (p20 CASP1, ****P < 0.0001) (Pro-GSDMD, ****P < 0.0001) (Cleaved GSDMD, ****P < 0.0001) (Pro IL-1B, ****P < 0.0001) (CD81, ****P < 0.0001) (L1CAM, **P = 0.0018). Error bars represent s.e.m.

Inflammasome activation and pyroptosis are widely reported to result in the release of membrane-bound EVs^29,40,41^. To determine if the plasma-borne NLRP3 we detected was contained within EVs and indicative of inflammasome activation, we isolated and analyzed EVs from plasma samples and compared the EV fraction with the soluble plasma fraction using SDS–PAGE (Fig. 3a). We readily confirmed the immunosorbent assay data (Tables 1–3) detecting NLRP3 in both healthy control and PD patients. Plasma-borne NLRP3 appeared exclusively in the CD81-positive EV fraction (Fig. 3a, b) indicative of pyroptosis-associated vesicular shedding. We found clear evidence of other pyroptosis-related proteins in the EV fraction in association with circulating NLRP3 including ASC, CASP1, and GSDMD, and Pro-IL-1B (Fig. 3). Based on our observation of multi-system cellular origins of NLRP3 in PD (Fig. 1, Supplemental Figs. 2 and 3) and previous reports by others of CNS-derived EVs in peripheral circulation^59,60^, we analyzed these same samples using a marker for EVs of nervous system origin, L1CAM^61^. As expected, L1CAM expression was only detectable in the EV fraction. We observed variable and sometimes high levels of expression of L1CAM within the eight plasma samples available for analysis (Fig. 3a, b). Based on these data, we can reasonably infer that the presence of NLRP3 protein in plasma is a surrogate for inflammasome activity and pyroptosis, regardless of disease state.

Plasma is a complex biofluid and any means of fractionating this matrix has the potential to increase the sensitivity of biomarker assays^62^. In forward looking studies, we re-optimized our electrochemiluminescent assay for the concentrated EV-protein fraction (Supplemental Fig. 5) and evaluated samples from our Dartmouth cohort (Table 4, Supplemental Fig. 6). Data from this smaller group did not withstand regression analysis, however, when analyzing the EV fraction, we noted a clear increase in the proportion of individuals [*n* = 42 of 162 (26%)] in whom we were able to detect plasma-borne NLRP3 compared with crude plasma samples [*n* = 25 of 162 (15%)]. When we considered NLRP3 as a binary variable, there was clearly a higher proportion of PD patients with detectable EV-borne NLRP3 [EVs: *n* = 31 (31% of PD)] compared with controls [EVs: *n* = 11 (18% of controls)] (*P* = 0.11), whereas the crude plasma NLRP3 levels did not differ by PD status (plasma: *n* = 17 (17% of PD) *n* = 8 (13% of controls) (*P* = 0.68). Further, the detectable concentration of NLRP3 was significantly higher in the EV fraction compared to its respective plasma sample (*P* = 0.0411) (Supplemental Fig. 6). These data suggest that future studies designed to isolate and further evaluate inflammasome-related EVs may provide an inroad towards PD stratification and the discovery of plasma-borne biomarkers of inflammation.

**Table 4:** NLRP3 is higher in the EV fraction than crude plasma in the DHMC cohort.

		Control	PD	*P* univariate
		*N* = 61	*N* = 101	
Age (mean (SD))		67.15 (9.50)	69.83 (8.13)	0.058
Sex = Male (%)		13 (21.3)	71 (70.3)	<0.001
BMI (mean (SD))		27.45 (4.39)	25.88 (4.24)	0.059
Age of onset (mean (SD))		n/a	65.23 (9.55)	n/a
UPDRS score (mean (SD))		n/a	26.23 (14.14)	n/a
DA replacement = Yes (%)		n/a	80 (79.2)	n/a
DA agonist = Yes (%)		n/a	27 (26.7)	n/a
DA anti-degradation = Yes (%)		n/a	31 (30.7)	n/a
NSAID = Yes (%)		10 (16.4)	30 (29.7)	0.086
	Acetaminophen^a^	5 (8.2)	20 (19.8)	0.079
	Aspirin	17 (27.9)	40 (39.6)	0.18
Plasma NLRP3	≤0.1 ng/mL	53 (86.9)	84 (83.2)	0.68
	0.1+ ng/mL	8 (13.1)	17 (16.8)	
EV NLRP3	≤0.1 ng/mL	50 (82.0)	70 (69.3)	0.11
	0.1+ ng/mL	11 (18.0)	31 (30.7)	

^a^Non-NSAID, non-aspirin pain reliever.

## Discussion

Our studies build on a growing body of evidence indicating a role for *NLRP3* in cell, animal, and human studies of neurodegenerative disease^17,27,31,63^. We have considered the secretory aspects of inflammasome activity and explored the potential that the release of cytosolic proteins observed during these pro-inflammatory processes^36,37,64,65^ may result in the detection of pyroptosis-related proteins in biofluids. In our studies focused on PD, we have confirmed that numerous cell types including microglia and MNC have the potential to contribute to an extracellular pool of pyroptosis-related proteins (Supplemental Figs. 2 and 3). These findings are consistent with multiple reports^27,31,52^ and a recent patient study showing elevation of inflammasome-related transcripts and increased protein levels of NLRP3, caspase-1, and IL-1β in peripheral blood mononuclear cells obtained from PD patients^66^. We have tested the prediction that inflammasome-related proteins circulated in PD in a clinical study and found that a subset of patients have readily detectable plasma-borne NLRP3 (Tables 1 and 2) and that high levels of plasma-borne NLRP3 are associated with PD (Tables 1–3). These patient studies that include histology and analysis of biofluids provide significant advances in our understanding of inflammasome activity in PD. Data suggest that inflammasome activity begins early during the degeneration of the mesencephalon (Fig. 1), is observable in multiple systems (Supplemental Figs. 2 and 3), and likely underlies a potentially rich source of serologic indicators associated with inflammation and PD progression (Table 3).

This study focused on establishment of a patient cohort and the development of a new assay to validate recent studies of the NLRP3 inflammasome in cells and animals while also providing proof-of-principle data to support the expansion of translational studies of the inflammasomes in PD. Data should be interpreted with caution at this stage as many limitations remain. There is a consensus that cells of the innate immune system release inflammasome-related proteins (Supplemental Figs. 2 and 3)^45,52,53^ and our data and others suggest that other cell types including ECs (Supplemental Fig. 2)^51^ and neurons (Supplemental Fig. 1)^19,46^ may also be capable of inflammasome-related secretory activities. Data presented here are not sufficient to infer cellular origins of circulating inflammasome-related proteins. Studies do suggest the potential of complex multi-cellular origins that range from neuronal to peripheral immune cell populations. New models designed to specifically identify the discrete contributions of these cell types to an extracellular pool of inflammasome-related proteins will aid in the determination of which cell types may be both critical, and therapeutically modifiable, in the context of PD. Our immunosorbent assay is highly specific (Fig. 2), however, it is unclear to what degree the steps we took to eliminate background impacted sensitivity, perhaps underlying the high percentage of patients in which we were unable to detect NLRP3. Current limitations of this and related assays will need to be overcome in order to improve sensitivity while managing the inherently confounding matrix effect associated with human biofluids. The large proportion of individuals found to have no detectable NLRP3 is of interest given the intense effort to better stratify PD using clinically applicable metrics. Planned studies will determine the robustness of this remarkable distinction, capitalize on our identification of potentially useful fractionating strategies (Table 4, Supplemental Fig. 6), and provide longitudinal analyses of individual patients to determine the temporal variability of circulating inflammasome-related proteins in PD.

PD is slowly progressive, with many patients living with the disease for decades. This presents a limitation with regards to obtaining and analyzing brain tissues representing “early-stage” PD. In an effort to overcome this longstanding challenge, we leveraged the biorepository at Dartmouth-Hitchcock Medical Center to identify patients characterized post-mortem as having nigral cell loss in the absence of clinical symptomology. We reasoned that these cases, regardless of whether individuals may have eventually developed PD, could serve as surrogates for analysis of early stage nigral degeneration. Upon further analysis, these individuals indeed had readily observable reductions in the number of pigmented neurons in the SNpC (Fig. 1) with numbers intermediate compared with tissues obtained from healthy controls and histopathologically confirmed PD patients. Close pathologic inspection of these tissues revealed further evidence of neurodegeneration. We observed a large number of pigment-laden MAC in the nigral loss and PD subsets, characteristic of parkinsonian neurodegeneration^67,68^, and indicative of an immune response to dopaminergic cell death (Supplemental Fig. 7). In the sections we evaluated, we observed frequent Lewy Bodies in PD cases and were unable to identify obvious Lewy Body pathology using either H&E or α-synuclein immunostaining in the nigral loss tissues. Taken together, these tissues provide an interesting resource for evaluation of parkinsonian histopathologies occurring in the absence of clinical symptomology. Elevation of NLRP3 inflammasome expression and the formation of ASC “specks” in tissues from individuals with nigral degeneration not previously identified to have clinical symptoms of PD indicate that inflammasome activation is likely an early event in the progression of PD.

There is clear evidence supporting a role for inflammation in a wide variety of neurodegenerative diseases^69,70^, including PD^71,72^. Inflammasomes are gaining attention due to their ability to respond to not only pathogen-associated molecular patterns (PAMPs), but also danger-associated molecular patterns (DAMPs)^30^, such as mitochondrial ROS^73^, misfolded proteins^24,74^, lysosomal dysfunction^75^, etc., which are common to not only PD^76–78^, but AD^79,80^ as well as other neurodegenerative disorders^81,82^. Unlike apoptosis and other more frequently studied cell death programs related to PD^83^, pyroptosis is an important aspect of inflammasome activity that is not well-characterized in brain disorders. Apoptosis relies on interactions between caspases including caspase-3, caspase-8, and caspase-9, and the Bcl family of proteins resulting in an immunologically tolerable cell death^34,84^. Pyroptosis has several distinct qualities including a reliance on caspase-1, vesicular shedding, and the cleavage of proteins like GSDMD that create pores in the cell membrane^15^. This results in a highly inflammatory form of cell death that goes beyond the release of pro-inflammatory cytokines to include the release of other cytosolic proteins. Consistent with multiple reports, our study confirms that MNC and microglia actively release pyroptosis-related proteins into the extracellular space^20,21,85,86^. We have extended these previous studies, finding pyroptosis-related proteins in human plasma samples and an association between elevated levels of circulating NLRP3 and PD. Further evaluation of plasma compartmentalization of these circulating proteins supports a model in which pyroptosis occurs on an ongoing basis in living patients by showing that pyroptosis-related proteins are contained within EVs, some of which bear markers of neuronal origin. L1CAM is a cell adhesion molecule found on the surface of neurons and neuronal-derived EVs^61^. We saw variable expression of L1CAM in the EV fraction across the eight patients analyzed (Fig. 3), suggesting neuronal EVs contribute to the pool of peripherally obtained EVs to differing degrees. We were unable to confirm the exact origin of the inflammasome-containing EVs from the heterogenous pool we obtained, however, the current literature predicts that they may be derived from many cells types capable of secreting EVs following inflammasome activation including peripheral immune^54^ and CNS cells^29^. While the finding of high levels of L1CAM in EVs obtained from several individuals is intriguing, the specificity of L1CAM remains a subject of debate^87,88^ and its presence cannot distinguish between peripheral and central neurons. Unbiased screens aided by identification and enrichment for EV markers specific for inflammasome-related vesicles bear the potential to both identify cellular and tissue origins of inflammasome activity and also characterize the entirety of the inflammasome-related secretome.

These studies are consistent with evidence of extracellular ASC specks present within the peritoneal space after inflammasome activation in mice^89^ as well as plasma-borne inflammasome specks in the blood of patients with active CAPS^54^. Key questions remain related to the timeline and sequence of pyroptotic inflammasome activation, cell swelling and rupture, and cell death. While resolution of inflammation and cell survival is observed in the innate immune system^90^, evidence in models of sepsis clearly indicate the potential for cell rupture in circulating MNC^91^. To our knowledge, the process of cell rupture in microglia is not well defined. It is interesting to consider the possibility that in PD and similar disorders, microglial pyroptotic mechanisms may underlie important processes beyond the chronicity of inflammation, including enhanced oxidative stress, the propagation of pathologic protein conformers, and neurotoxicity. In this context, whether or not cells release cytosolic contents via exocytosis, leakage through pores, or rupture may not matter as the process provides a platform for discovery of unexpected intracellular indicators and pro-inflammatory stimuli existing in an easily isolated sub-fraction of a readily accessible biofluid. Further evaluation of the pyroptosis-associated secretome in both plasma as well as cerebrospinal fluid has a high likelihood of identifying biomarkers and pathways of interest for understanding age-related neurodegenerative disorders like PD, as well as a panoply of other inflammation-related diseases.

An exciting aspect of recent advances in the analysis of inflammasomes in human diseases is the growing effort to develop small molecules designed to interrupt inflammasome activity. Gordon and others provide compelling evidence of a therapeutic effect resulting from treatment with anti-NLRP3 inflammasome drugs in multiple mouse models of PD^27,33,92^. Further reports suggest potent anti-inflammatory activity for inflammasome inhibitors in animal models of other human inflammatory diseases, such as gout^93^ and arthritis^94^. Our study supports efforts to target inflammasomes in PD based on our recognition that use of certain anti-inflammatory drugs are negatively correlated with plasma levels of NLRP3 (Table 2). Many studies have suggested the neuroprotective effect of NSAID use against developing PD^10,11^. Although not statistically significant in our study, we observed a lower proportion of patients with elevated NLRP3 (*p* = 0.11) among ibuprofen users, an NSAID well associated with a reduced risk of PD^10^. These data are consistent with previous reports suggesting certain NSAIDs are capable of inhibiting NLRP3^95^ and studies reporting mechanistic links between one major NSAID target, COX2, and the NLRP3 inflammasome^96,97^. Based on the frequent reporting of “over-the-counter” anti-inflammatory drug use by PD patients, our data indicating elevated NLRP3 in PD is likely an underrepresentation of the actual relationship between circulating pyroptosis-related proteins and PD. While our recruiting efforts and partnership with the Harvard Biomarker Study resulted in an association detected using rigorous multivariate analysis, we anticipate ongoing efforts to stratify patient populations based on medication, clinical history, and occupation will strengthen this association. Additionally, based on our findings that pyroptosis-related proteins: (1) exist in the easily precipitated EV fraction, (2) are detected at higher concentrations within the EV fraction, and (3) are detected with greater frequency in the EV fraction, we suggest that plasma fractionation at the time of collection may be a viable strategy to improve our ability to detect and utilize circulating pyroptosis-related proteins as method of disease stratification. Forward-looking studies planned that include continued optimization, validation of new related markers, and prospective and comparative studies designed to evaluate PD patients who are, and are not, utilizing certain medications have a high likelihood of yielding new information regarding the relationship between plasma-borne pyroptosis-related proteins and PD. The findings presented here and the future studies described are of high significance as new treatments designed to block inflammasome activity enter clinical trials. It is anticipated that plasma-borne pyroptosis-related proteins will play a key role in determining the efficacy of these important efforts to combat PD and related disorders.

## Methods

### Histology

De-identified post-mortem tissues were obtained from appropriately consented decedents with studies approved and overseen by the Committee for Protection of Human Subjects, Dartmouth-Hitchcock Medical Center (DHMC). A list of all autopsies conducted at DHMC from 2013 to 2017 was obtained from the Pathology Department. After receiving approved access to DHMC’s Medical Record database (eD-H), neuropathology reports (*n* = 335) for all conducted autopsies during the 2013–2017 time period were reviewed. Upon identifying cases with banked mesencephalic tissues (*n* = 173), the following information was collected from each file: age, sex, neurologic disease status, pathologic cause of death, and description of the substantia nigra. This process identified individuals with PD (*n* = 30), amyotrophic lateral sclerosis (ALS) (*n* = 4), Alzheimer’s disease (AD) (*n* = 11), and no neurologic disorder (*n* = 128). Individuals with no neurologic disorder (*n* = 128) had neuropathology reports describing the SNpC as either having no abnormal findings (*n* = 104) or nigral neuron loss (*n* = 24). Immunostaining was performed on 5 μM formalin-fixed paraffin-embedded (FFPE) histologic sections using the automated Leica Bond-Max platform as we previously reported^19^. Anti-NLRP3 (D4D8T, Cell Signaling Technology, Danvers, MA; 1:200) and anti-ASC (AL177, Adipogen, San Diego, CA; 1:500) primary antibodies were detected using a post-primary biotin-free alkaline phosphate polymer and fast red chromogen (Leica Biosystems, Buffalo Grove, IL). For immunofluorescent labeling, 5 μM FFPE histologic slides were deparaffinized, and epitope retrieval was conducted using citrate buffer. After blocking in 1% BSA/TBS, slides were incubated with primary antibodies overnight at 4 °C in blocking buffer: NLRP3 (D4D8T, Cell Signaling Technology, Danvers, MA; 1:200), TH (MAB318, MilliporeSigma, Burlington, MA; 1:500), GFAP (3670, Cell Signaling Technology, Danvers, MA; 1:800), and/or IBA1 (NB100-1028, Novus Biologicals, Centennial, CO; 1:500). Slides were incubated with secondary antibodies raised in donkey (secondary Alexa Fluor 488 ab150061, Alexa Fluor 488 ab150129, and Alexa Fluor 555 ab150130, Abcam, Cambridge, United Kingdom) for 1 h protected from light and counterstained with Hoechst dye (3342, Thermo Fisher Scientific, Waltham, MA). Labeled sections were mounted with PermaFlour (TA-030-FM, Thermo Fisher Scientific, Waltham, MA) and imaged using confocal microscopy.

### Cell culture

All cells were maintained in a humidified 37 °C incubator with 95% O_2_ and 5% CO_2._ THP-1 cells (ATCC, TIB-202) were cultured in RPMI 1640 with L-glutamine (10-040-CV, Corning, Corning, NY) containing 10% fetal bovine serum and 1% penicillin/streptomycin. Primary mixed glia cultures were established from whole brains obtained on postnatal day 1–3 from WT and *Nlrp3*^*−/−*^ mice of mixed sexes. Whole brains were digested and triturated in 400 μL of Accutase (A6964, MilliporeSigma, Burlington, MA). Brain cell suspensions were passed through a 70 μM filter and plated in tissue culture dishes, split 1:3 after 4–5 days and allowed to expand for an additional 4–5 days prior to treatment. Primary mixed glial cells were cultured in Dulbecco’s modified Eagle’s medium (DMEM) with 10% fetal bovine serum and 1% penicillin/streptomycin. NLRP3 priming and activation was conducted in serum-free media as previously reported^41^ consisting of 6 h incubation with 1 ng/mL LPS followed by 30 min incubation with 10 μM nigericin sodium salt (N7143, Sigma-Aldrich, St. Louis, MO). Conditioned media was collected and centrifuged at 12,000 RPM for 30 min at 4 °C to remove cellular debris and frozen at −80 °C until total protein precipitation and analysis via SDS–PAGE.

### Analysis of conditioned media and immunoprecipitation

Total protein was precipitated from media following treatment of cell cultures as previously described^24^. The pellet was resuspended in 50 μL of 4× NuPAGE™ LDS sample buffer (NP0008, Thermo Fisher Scientific, Waltham, MA) diluted to 1× with lysis buffer (150 mm NaCl, 50 mm Tris, 1% Triton X-100) containing 1× phosphatase inhibitor cocktail 2 (P5726, Sigma-Aldrich, St. Louis, MO) and 1× Complete Mini Protease Inhibitor Cocktail (11836153001, Sigma-Aldrich, St. Louis, MO). The samples were sonicated briefly at 20 AMP, 0.02% beta-mercaptoethanol (BME) added, and boiled for 10 min at 100 °C. Gel fractionation was conducted using pre-cast 4–12% Bis–Tris protein gels and MES/SDS running buffer (Thermo Fisher Scientific, Waltham, MA). Gels were transferred and immunoblotted using standard techniques as previously described^98^. The following antibodies were used for the detection of inflammasome-related proteins in cell culture supernatant: anti-NLRP3 (AG-20B-0014, Adipogen, San Diego, CA; 15101, Cell Signaling Technology, Danvers, MA), anti-CASP1 (p20) (AG-20B-0048B, Adipogen, San Diego, CA), anti-CASP1 (p10) (AG-20B-0042, Adipogen, San Diego, CA), anti-GSDMD (93709, Cell Signaling Technology, Danvers, MA), anti-ASC (AG-25B-0006, Adipogen, San Diego, CA; 67824, Cell Signaling Technology, Danvers, MA), and anti-IL-1B (NB600-633, Novus Biologicals, Centennial, CO; AF-401, R&D Systems, Minneapolis, MN).

For antibody pair validation, immunoprecipitation assays used the same capture and detection antibodies from the electrochemiluminescent assays as the pull-down and blotting antibodies, respectively. The antibodies used for the human EV lysates were the same as those above used for the detection of inflammasome-related proteins in THP-1 cell culture supernatant. For immunoblots performed, all blots were derived from the same experiment and processed in parallel.

### Immunoassay development

Electrochemiluminescent NLRP3 assay was developed using the ELISA Conversion Pack I (uncoated plates) (#K15A01-1) obtained from Meso Scale Discovery (MSD) (Rockville, MD). For determination of a working antibody pair, a 2 μg/mL dilution of capture antibodies in 1× phosphate-buffered saline (PBS) (21-040-CV, Corning, Corning, NY) was adsorbed, blocked with 3% w/v Blocker A (MSD, Rockville, MD) in PBS and washed three times (3×) with 200 µl PBS + 0.05% Tween 20 (PBS-T). Standard curves were created using two-fold serial diluted recombinant NLRP3 protein (LS-G36168, LifeSpan BioSciences, Seattle, WA) across a range of 50–0.78 ng in a 1% Blocker A /PBS. After a two-hour incubation on an orbital shaker (180 rpm), the samples were removed, the wells were washed 3× with 200 µl PBS-T, and 25 µL of a 1 μg/mL series of potential detection antibodies diluted in 1% Blocker A/PBS solution was examined in combination with each capture antibody. Species-specific SULFO-TAG antibodies (MSD, Rockville, MD) were used to generate luminescence and the assay plates were read using an MSD Sector Imager 2400 plate reader.

Promising antibody pairings were further analyzed in immunoprecipitation assays as previously reported. Briefly, recombinant NLRP3 protein was added to human plasma samples, pre-cleared using 10 µl of pre-washed Protein G Agarose beads, and 2 μg/mL of capture antibody was added to immunoprecipitate each sample. Immunoprecipitate was resuspended in 1× diluted 4× NuPAGE™ LDS sample buffer (NP0008, Thermo Fisher Scientific, Waltham, MA) with 0.02% BME, subject to SDS–PAGE and immunoblotting analysis using the associated detection antibody. A single antibody pair was found capable of detecting recombinant NLRP3 protein: capture, Cryo 2 (AG-20B-0014-C100, Adipogen, San Diego, CA), detection, D4D8T (15101, Cell Signaling Technologies, Danvers, MA).

The antibody pair described above, capable of detecting serially diluted spiked recombinant protein in BSA with minimal background and identifying a single band in immunoprecipitation assays, was then used to optimize the plasma matrix dilution. For this assay, the capture antibody was biotinylated using the EZ-Link™ Sulfo-NHS-Biotin, No-Weigh™ Format (A39256, Thermo Fisher Scientific, Waltham, MA) and analysis was conducted using the more sensitive MSD GOLD Small Spot Streptavidin 96-well plates with a capture antibody concentration of 0.5 μg/mL. Assays were conducted using serially diluted recombinant protein as well as serially diluted plasma to determine the optimal plasma dilution for NLRP3 detection. Optimal plasma dilution was determined by comparing the *r*^2^ values of the linear regression curves generated from each plasma dilution as well as the level of background, using biotinylated mouse IgG as a control for non-specific binding. For detection of NLRP3, a 1:16 fold (6.25%) plasma concentration yielded the highest sensitivity with the lowest background. Wash stringency analysis, with buffers of increasing stringency, was performed in order to confirm the method of plate washing most effective at reducing non-specific-binding while maintaining analyte recovery. The least stringent washing method, of 4× with PBS-T (PBS + 0.05% Tween 20) was found superior to washes with increasing salt content.

Similar methods were utilized for re-optimization of the assay for NLRP3 detection from protein-rich EV preps. Briefly, various dilutions of capture antibody were tested for optimal NLRP3 detection in samples spiked with recombinant NLRP3 protein finding a capture antibody concentration of 1 μg/mL to be most effective. Tests were performed in order to confirm assay compatibility assay with the EV diluent lysis buffer (150 mm NaCl, 50 mm Tris, 1% Triton X-100) containing 1× phosphatase inhibitor cocktail 2 (P5726, Sigma-Aldrich, St. Louis, MO) and 1× Complete Mini Protease Inhibitor Cocktail (11836153001, Sigma-Aldrich, St. Louis, MO). Optimal EV dilution was determined by comparing the recovery of spiked recombinant NLRP3 protein in various dilutions of EV prep, using biotinylated mouse IgG as a control for assay specificity and non-specific binding. Identical wash stringency analysis, as previously described, was performed for EV re-optimization finding again that washing 4× with PBS-T (PBS + 0.05% Tween 20) was most effective.

### Transcriptome analysis

Analysis of pyroptosis-related mRNA expression in the mouse brain was conducted using the publicly available Single Cell Portal maintained by the Broad Institute (Cambridge, MA). The dataset for aged mouse brain utilized was originally reported by Ximerakis et al. (2019)^51^.

### Plasma collection and processing

Patients over 21 years of age were enrolled and consented at the Dartmouth-Hitchcock Clinic (Lebanon, NH) within the Department of Neurology and plasma samples were obtained at the Dartmouth-Hitchcock Clinical Research Unit. Exclusion criteria for all participants included pregnant women, drug-induced parkinsonism, acute or chronic infection, traumatic injury within the last 90 days, exposure to corticosteroids within the last 90 days, or neoplastic disease (cancer). Exclusion criteria for control patients included history of neurologic disorder. Study enrollment began April 2017 and is ongoing. In addition to samples obtained at Dartmouth, plasma samples were generously provided by Dr. Clemens Scherzer, collected in the Harvard Biomarker Study (https://www.bwhparkinsoncenter.org/biobank/)^99,100^, Harvard Medical School, Brigham and Women’s Hospital, and Massachusetts General Hospitals (Boston, MA).

Plasma processing, cryopreservation, and annotation were carried out in DartLab, the Immune Monitoring and Flow Cytometry Shared Resource at the Norris Cotton Cancer Center at Dartmouth, with NCI Cancer Center Support Grant 5P30 CA023108-37. Briefly, whole blood was collected in K_2_-EDTA tubes. For plasma processing, the K_2_-EDTA vacutainer was placed into a fresh 50 mL conical tube and centrifuged at 1125 × *g* for 10 min at RT. The vacutainer was removed from the 50 mL containment tube and carefully uncapped. Plasma was removed and placed into a fresh 15 mL conical tube and centrifuged at 1825 × *g* for 10 min at RT. After centrifugation, the plasma was aliquoted into 250 μL aliquots and frozen at −80 °C for future analysis or immediately processed (see section “Extracellular vesicle isolation” below).

### EV isolation

Total EVs were isolated from fresh drawn or frozen human plasma samples using the ExoQuick Plasma Prep with Thrombin kit (EXOQ5TM-1, System Biosciences, Palo Alto, CA) according to the manufacturer’s instructions. Four plasma fractions were generated for analysis: (1) crude, fresh drawn plasma centrifuged at 1500 × *g* for 15 min at 4 °C; (2) thrombin-cleared, crude plasma incubated with thrombin clearing agent for 5 min at RT and centrifuged at 10,000 RPM for 5 min; (3) soluble, top-fraction of thrombin-cleared plasma incubated with Exoquik reagent for 30 min at 4 °C and centrifuged at 1500×*g* for 30 min at 4 °C; and (4) EVs, bottom-fraction of thrombin-cleared plasma incubated with Exoquik reagent for 30 min at 4 °C and centrifuged at 1500 × *g* for 30 min at 4 °C. Once isolated, the EVs were lysed with ice-cold lysis buffer (150 mm NaCl, 50 mm Tris, 1% Triton X-100) containing 1× phosphatase inhibitor cocktail 2 (P5726, Sigma-Aldrich, St. Louis, MO) and 1× Complete Mini Protease Inhibitor Cocktail (11836153001, Sigma-Aldrich, St. Louis, MO) and sonicated at 20 AMP for a few seconds. All fractions were used fresh for SDS–PAGE analysis or frozen at −80 °C until analysis via MSD.

### Statistics

Immunochemistry and SDS–PAGE experiments were conducted with four and three independently collected biologic replicates, respectively, analyzed in technical triplicates, and all data expressed as mean ± SEM unless otherwise stated. Numeric data were derived from immunoblots using densitometry and relative protein levels were determined by normalizing to housekeeping protein β-actin. Paired data were analyzed using two-tailed *T*-tests. One-way ANOVAs were used for multiple comparisons corrected using a Tukey’s post-hoc test when appropriate. Linear regression was used to determine the extent of a relationship between two variables. Statistical significance was reached with a *p* < 0.05 for all analyses performed.

For patient plasma analysis, the continuous variables were levels of the various analytes obtained using the optimized immunosorbent assays described above. Univariate analyses were performed using chi-square tests for categorical variables and *t*-tests for continuous variables to identify factors associated with PD status or elevated NLRP3. Multivariable logistic regression models were then constructed to assess factors associated with PD status adjusted for covariates (e.g. age and sex).

### Study approval

All human subjects research was performed in accordance with the guidelines described in the Declaration of Helsinki. Informed written consent was obtained from all patients who participated in this study, and appropriate measures were utilized to minimize risk and ensure equitable opportunity for participation across the catchment area. Human studies conducted at Dartmouth-Hitchcock Medical Center have been reviewed, approved, and are overseen by The Institutional Review Board of Dartmouth College (CPHS#: STUDY00030209). All mouse experiments were conducted according to the ARRIVE guidelines and approved by the Institutional Animal Care and Use Committee (IACUC) at Dartmouth protocol entitled “*Animal Models of Neurodegenerative Diseases*” protocol #00002117.

### Reporting summary

Further information on research design is available in the Nature Research Reporting Summary linked to this article.

## Supplementary information

Supplemental Material_Full blots

Reporting Summary Checklist

## Data Availability

Mouse genomic data are publicly available at the Broad Single Cell Portal (https://portals.broadinstitute.org/single_cell), raw dataset. GEO: (https://www.ncbi.nlm.nih.gov/geo/query/acc.cgi?acc=GSE129788), original publication^51^.
