# Supplementary material for: Plasma-borne indicators of inflammasome activity in Parkinson’s disease patients
Source: NPJ Parkinsons Dis. 2021 Jan 4;7:2. doi: 10.1038/s41531-020-00147-6 (PMC7782812; doi:10.1038/s41531-020-00147-6)
Supplement: Supplementary file 1 — Supplemental Material_Full blots [file 41531_2020_147_MOESM1_ESM.docx]

**Supplemental Material**

Supplemental Table 1. Post-mortem neuropathological descriptions of mesencephalic tissues obtained from control subjects noted to have an unremarkable SNpC, control subjects noted to have loss of SNpC neurons, and clinically confirmed PD patients.

| **Case** | **Age** | **Sex** | **Tissue** | **Neuropathology Description** |
| --- | --- | --- | --- | --- |
| C-1 | 69 | F | SN | Midbrain is unremarkable. |
| C-2 | 76 | M | SN | SN normally pigmented. |
| C-3 | 71 | M | SN | Normally pigmented SN. |
| C-4 | 75 | F | SN | Normally pigmented SN. |
| C-5 | 88 | F | SN | Normally pigmented SN. |
| C-6 | 55 | M | SN | Midbrain unremarkable. |
| NL-1 | 71 | F | SN | Mild neuronal loss in the SN; one of the colliculi shows extensive background gliosis. |
| NL-2 | 64 | M | SN | Mild neuronal loss in the SN; solitary neurofibrillary tangle in the locus coeruleus. |
| NL-3 | 77 | M | SN | Minor neurodegenerative changes in SN, no specific pathological changes. |
| NL-4 | 65 | F | SN | Mild neurodegenerative changes; neuronal loss in the SN. |
| NL-5 | 70 | M | SN | Minor loss of SN neurons. No other pathologic changes. |
| NL-6 | 74 | M | SN | Mild loss of neurons in SN. |
| PD-1 | 71 | M | SN | Neuronal loss, background gliosis, axonal spheroid forms in neurons with Lewy bodies in the SN |
| PD-2 | 85 | M | SN | Sections of mesencephalon with extensive neuron loss in SN; Lewy bodies found in background of gliosis and microglial activation. |
| PD-3 | 76 | M | SN | Melanin containing macrophages present; microglial activation and gliosis are also present; extensive, near total, loss of neurons. |
| PD-4 | 74 | M | SN | Extensive loss of SN neurons. Axonal spheroids in the SN. Occasional globose neurofibrillary tangle and Lewy bodies are identified. |
| PD-5 | 63 | M | SN | Sections of mesencephalon show loss of neurons in SN. Background gliosis and Lewy body formations. |
| PD-6 | 80 | M | SN | Extensive neuronal loss and gliosis in SN. Lewy bodies present in some of remaining neurons; loss of pigmentation. |


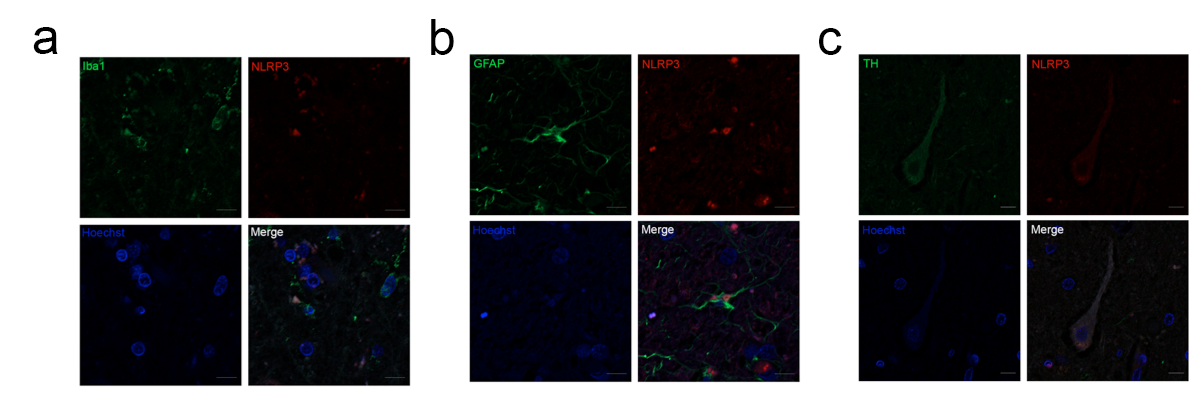
Supplemental Figure 1. NLRP3 co-localization within cells of the central nervous system. Adjacent histologic section to those evaluated in Figure 1 were analyzed using co-immunolabeling and confocal microscopy within the degenerating mesencephalon of PD patients. NLRP3 expression (red) was identified in IBA1-immunoreactive microglia (a, green), in close proximity to GFAP-immunoreactive astrocytes (b, green), and TH-immunoreactive neurons (c, green). Scale bars represent 10 μm.

**
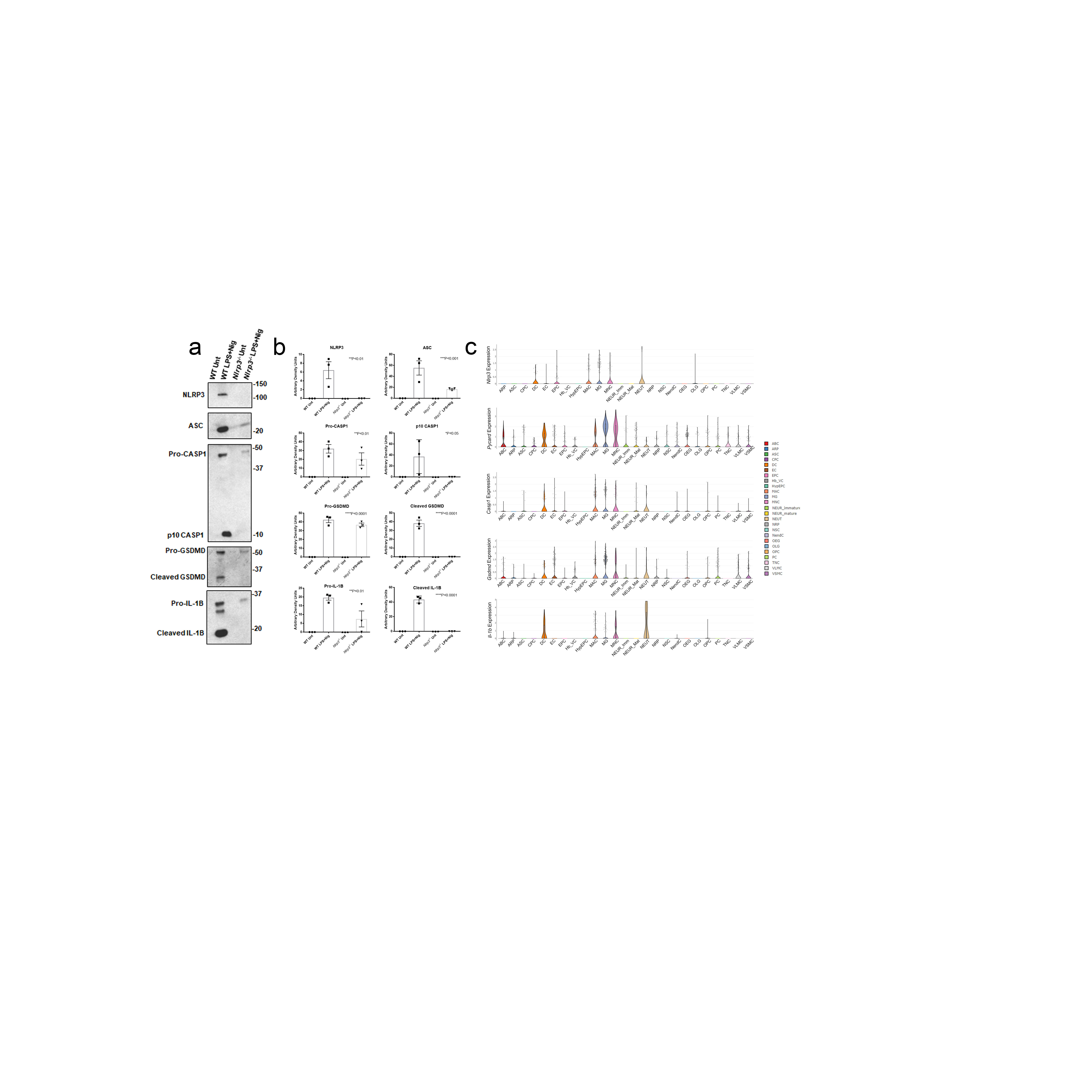
**

Supplemental Figure 2. Inflammasome-related proteins in cells of the central nervous system. (a) Primary mixed glial cultures from C57BL/6 wild-type (WT) (n=3) and *Nlrp3^-/-^* (n=3) mice were enriched for microglia. Cultures were treated with 1 μg/mL LPS for 6 hours followed by 10 μM nigericin-treatment for 30 minutes. Conditioned media was collected and total protein was methanol-precipitated and analyzed using SDS-PAGE and immunoblotting for inflammasome-related proteins of interest using anti-NLRP3, anti-ASC, anti-CASP1, anti-GSDMD, and anti-IL-1B antibodies. (b) Levels of inflammasome-related proteins were increased upon LPS and nigericin treatment in WT cultures compared to treated *Nlrp3^-/-^* cultures. All statistical analyses were performed using one-way ANOVAs with multiple comparisons across three biological replicates (NLRP3, ** *P* <0.01) (ASC, *** *P* <0.001) (Pro-CASP1, ** *P* <0.01) (p10 CASP1 * *P* <0.05) (Pro-GSDMD, **** *P* <0.0001) (Cleaved GSDMD, **** *P* <0.0001) (Pro IL-1B, ** *P* <0.01) (Cleaved IL-1B, **** *P* <0.0001). Error bars in b represent s.e.m. (c) A publicly-available single-cell RNASeq database of the aged mouse brain [51] was interrogated to determine which cell types of the CNS expressed inflammasome-related transcripts. Microglial (MG) cells were observed to express *Nlrp3*, *Pycard*, *Casp1*, as well as *GsdmD* and *IL-1b*. Dendritic cells (DCs), endothelial cell (ECs), monocytes (MNC), and macrophages (MAC) present in aged mouse were also observed to express the core components of the NLRP3 inflammasome. Cell abbreviations: ABC: Arachnoid barrier cells, ARP: Astrocyte-restricted precursors, ASC: Astrocytes, CPC: Choroid plexus epithelial cells, DC: Dendritic cells, EC: Endothelial cells, EPC: Ependymocytes, Hb-VC: Hemoglobin-expressing vascular cells, HypEPC: Hypendymal cells, MAC: Macrophages, MG: Microglia, MNC: Monocytes, NEUR_imm: immature Neurons, NEUR_mat: mature Neurons, NEUT: Neutrophils, NRP: Neuronal-restricted precursors, NSC: Neural stem cells, NendC: Neuroendocrine cells, OEG: Olfactory ensheathing glia, OLG: Oligodendrocytes, OPC: Oligodendrocyte precursor cells, PC: Pericytes, TNC: Tanycytes, VLMC: Vascular and leptomeningeal cells, and VSMC: Vascular smooth muscle cells.

**
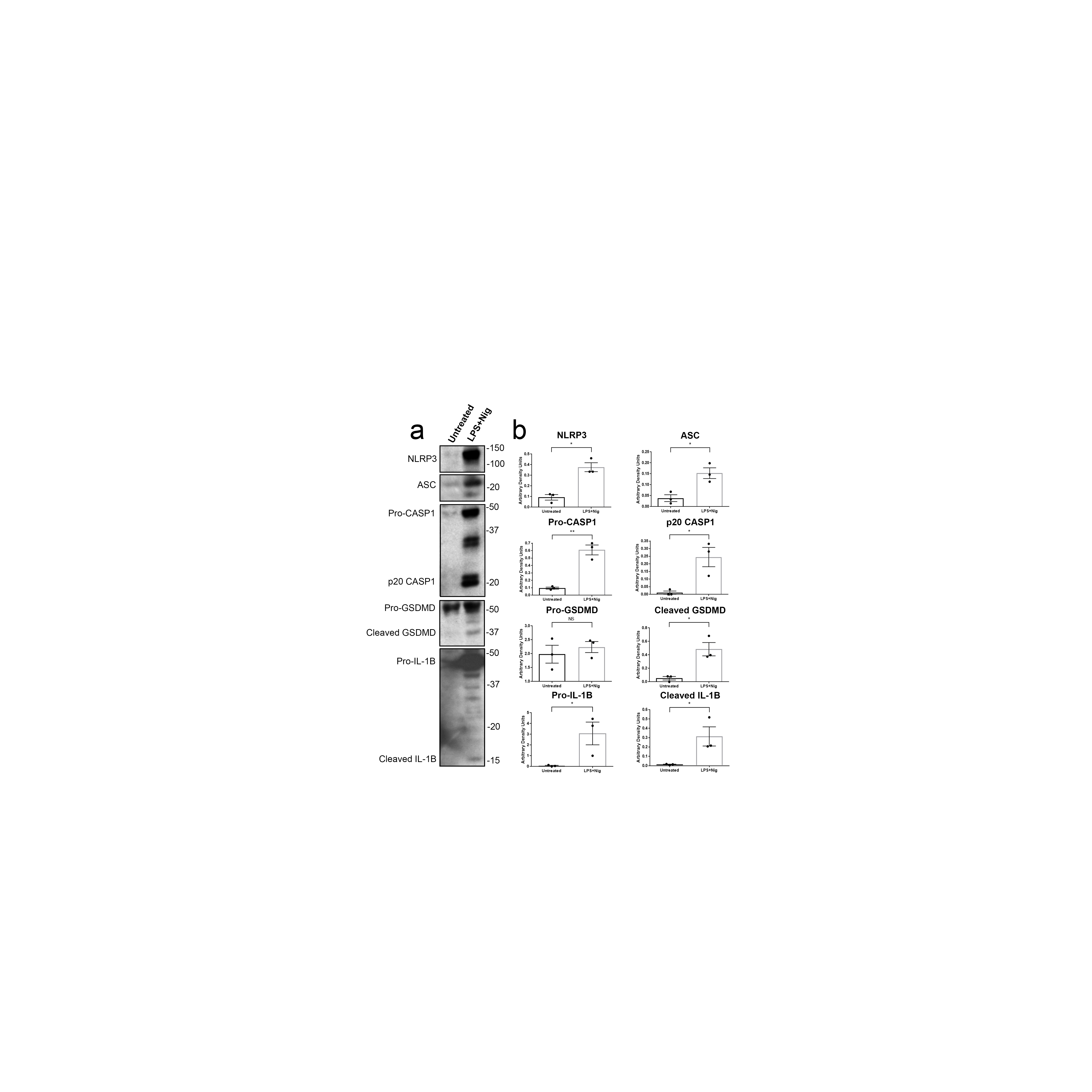
**

Supplemental Figure 3. Inflammasome-related proteins in THP-1 cells. (a) THP-1 monocytes were treated with 1 μg/mL LPS for 6 hours followed by 10 μM nigericin-treatment for 30 minutes. Conditioned media was collected and total protein was methanol-precipitated and analyzed using SDS-PAGE and subsequent immunoblotting for inflammasome-related proteins of interest using anti-NLRP3, anti-ASC, anti-CASP1, anti-GSDMD, and anti-IL-1B antibodies. (b) Levels of the majority of inflammasome-related proteins were increased upon LPS and nigericin treatment compared to untreated cultures where the presence of these proteins was typically either significantly reduced or not detectable. All statistical analyses were performed using unpaired Students t-tests across three biological replicates (NLRP3, ** P = 0.0046) (ASC, * P = 0.0170) (Pro-CASP1, ** P = 0.0016) (p10 CASP1 * P = 0.0220) (Pro-GSDMD, ns P = 0.5342) (Cleaved GSDMD, * P = 0.037) (Pro IL-1B, * P = 0.0463) (Cleaved IL-1B, * P = 0.0426). Error bars in b represent s.e.m.

Supplemental Table 2. Antibody pairs tested for use in MSD assay.

| **Analyte** | **Capture Antibody** | **Detection Antibody** | **Diluent** | **r^2^** |
| --- | --- | --- | --- | --- |
| NLRP3 | R&D Systems MAB7578 | Abcore AC21-0966-07 | 1% BSA | 0.5847 |
| NLRP3 | Adipogen Cryo 2 | Abcore AC21-0966-07 | 1% BSA | 0.9040 |
| NLRP3 | Cell Signaling D4D8T | Abcore AC21-0966-07 | 1% BSA | 0.8924 |
| NLRP3 | R&D Systems MAB7578 | Abcam ab4207 | 1% BSA | 0.1273 |
| NLRP3 | Cell Signaling D4D8T | Abcam ab4207 | 1% BSA | 0.0009 |
| NLRP3 | Adipogen Cryo 2 | Abcam ab4207 | 1% BSA | 0.0154 |
| NLRP3 | R&D Systems MAB7578 | Adipogen Cryo 2 | 1% BSA | 0.2066 |
| NLRP3 | Cell Signaling D4D8T | Adipogen Cryo 2 | 1% BSA | 0.0377 |
| NLRP3 | R&D Systems MAB7578 | Cell Signaling D4D8T | 1% BSA | 0.8591 |
| NLRP3 | Adipogen Cryo 2 | Cell Signaling D4D8T | 1% BSA | 0.9894 |

**
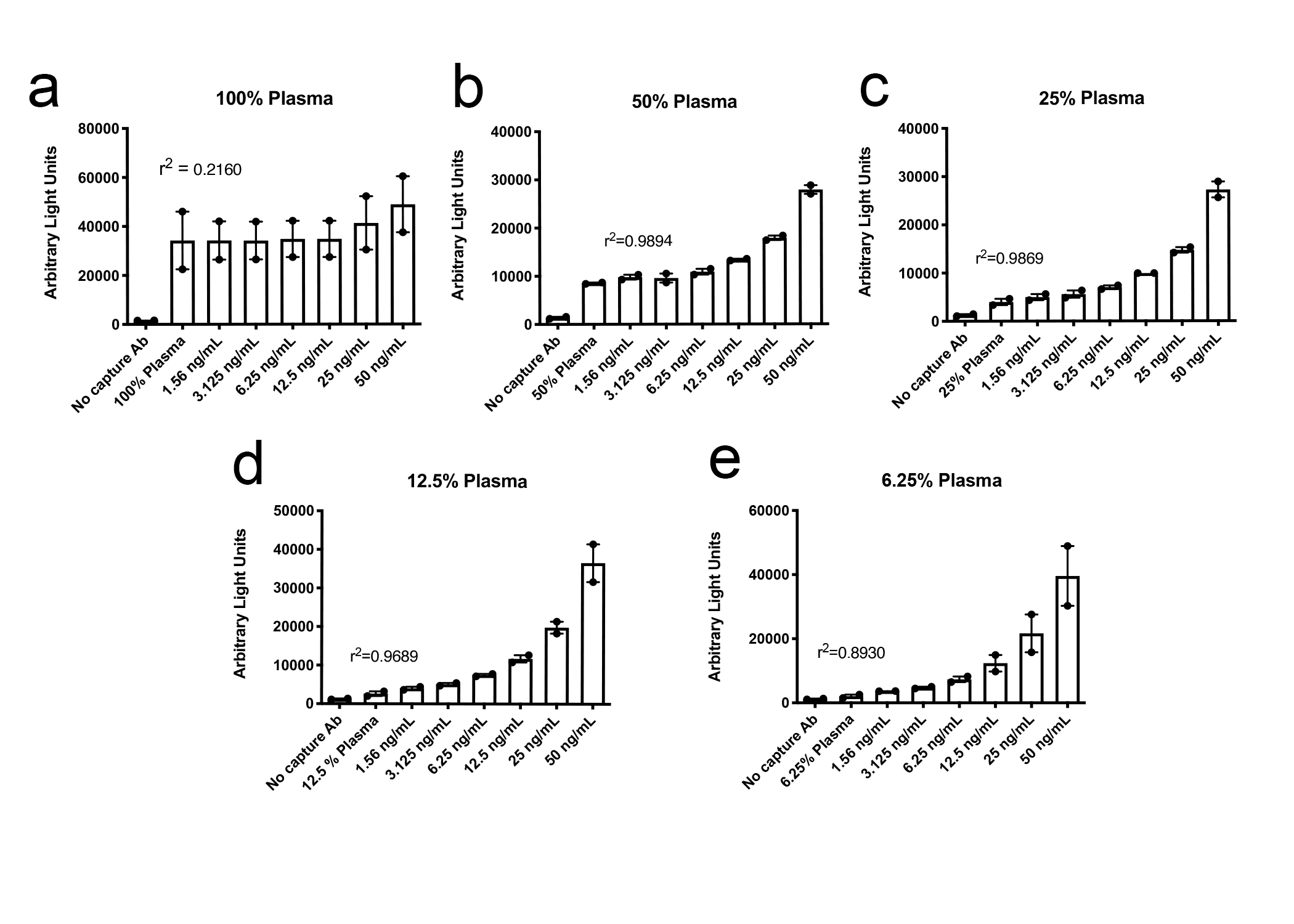
**

Supplemental Figure 4. Determining an optimal plasma dilution for the detection of recombinant NLRP3 protein. Once the antibody pair was determined, the capture antibody was biotinylated and assay conditions were optimized using streptavidin-coated 96-well electrode-containing immunosorbent plates. Serial two-fold dilutions of NLRP3 recombinant protein (RP) (50 ng/mL – 1.5625 ng/mL) were spiked into serial two-fold dilutions of plasma (100% – 1.5625%) in order to determine the optimal level of plasma dilution for protein detection. An optimal dilution of 6.25% (1:16 dilution) was found to detect NLRP3 RP (r^2^ = 0.8930) with minimal background. All statistical analyses were performed by linear regression, n=2 technical replicates. Error bars represent s.e.m.


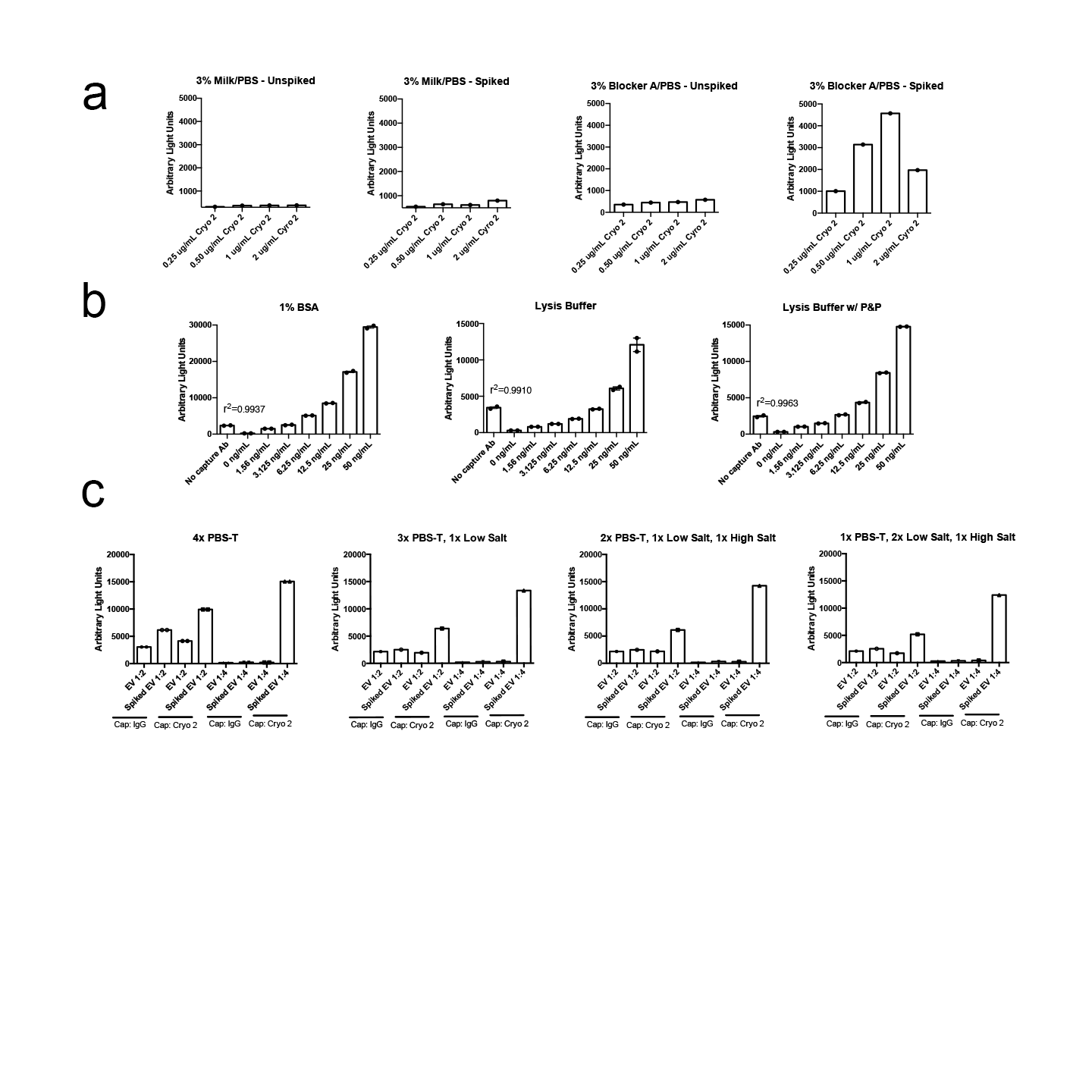


Supplemental Figure 5. NLRP3 assay re-optimization for EV analysis. (a) Various blocking agents were tested in order to achieve maximum background reduction, with 3% Blocker A/PBS (Meso Scale Diagnostics (MSD), Rockville, MD) allowing for relatively low background without masking analyte detection completely. Within these analyses, samples, with or without NLRP3 RP, were tested with various capture antibody concentrations in order to find optimal detection with low non-specific binding. A capture antibody concentration of 1 μg/mL was discovered to most optimally detect NLRP3 RP and was used for EV analysis. (b) Various diluents were tested in order to confirm assay compatibility with EV diluent buffer (lysis buffer (150 mm NaCl, 50 mm Tris, 1% Triton X-100) containing 1X phosphatase inhibitor cocktail 2 (P5726, Sigma-Aldrich, St. Louis, MO) and 1x Complete Mini Protease Inhibitor Cocktail (11836153001, Sigma-Aldrich, St. Louis, MO)). The assay was found to be compatible with EV diluent buffer and thus the standard curve was diluted in EV diluent buffer for all EV analyses. (c) Optimal EV dilution was determined by comparing the recovery of spiked recombinant NLRP3 protein in various dilutions (1:2, 1:4) of EV prep, using biotinylated mouse IgG as a control for assay specificity and non-specific binding. A dilution of 1:4 was found most optimal, with low background and non-specific binding to biotinylated mouse IgG. Within these studies, wash stringency analysis, with buffers of increasing salt concentration, was performed in order to confirm the method of plate washing most effective at reducing background while maintaining analyte recovery. The least stringent washing method, of 4x with PBS-T (PBS + 0.05% Tween 20) was found superior to washes with higher salt concentrations.


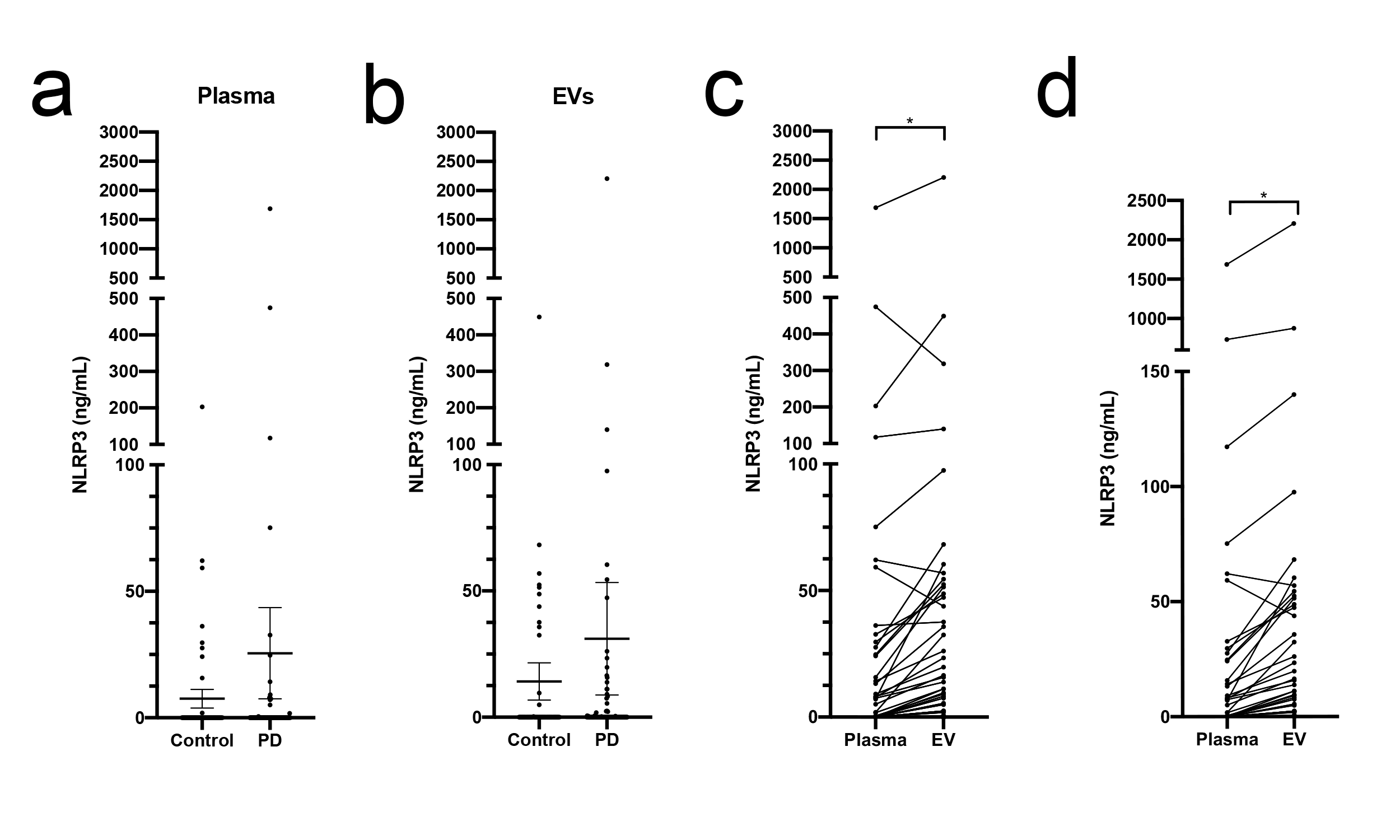
Supplemental Figure 6. Extracellular vesicles provide a rich source of plasma-borne NLRP3. (a) Extracellular vesicles (EVs) were isolated from all frozen plasma samples collected at the DHMC site and analyzed using the assay re-optimized for EV detection. Of the 162 plasma samples analyzed, NLRP3 was detectable in 25 samples. (b) The EV fractions of identical plasma samples were analyzed, finding 42 EV samples above the lower limit of detection. (c) In all samples where NLRP3 was detectable, the concentration was significantly higher in the EV fraction compared to its whole plasma counterpart (paired, one-tailed t-test, P = 0.0411). In some instances, NLRP3 was undetectable in the respective plasma sample but became detectable in the isolated EV fraction. (d) After removing three patients with non-PD related inflammatory co-morbidities, including inflamed meninges, nonalcoholic fatty liver disease, and glenohumeral arthritis, the concentration of NLRP3 was still significantly higher in the EV fraction compared to its whole plasma counterpart (paired, two-tailed t-test, P = 0.0443). Error bars in a and b represent s.e.m.

**
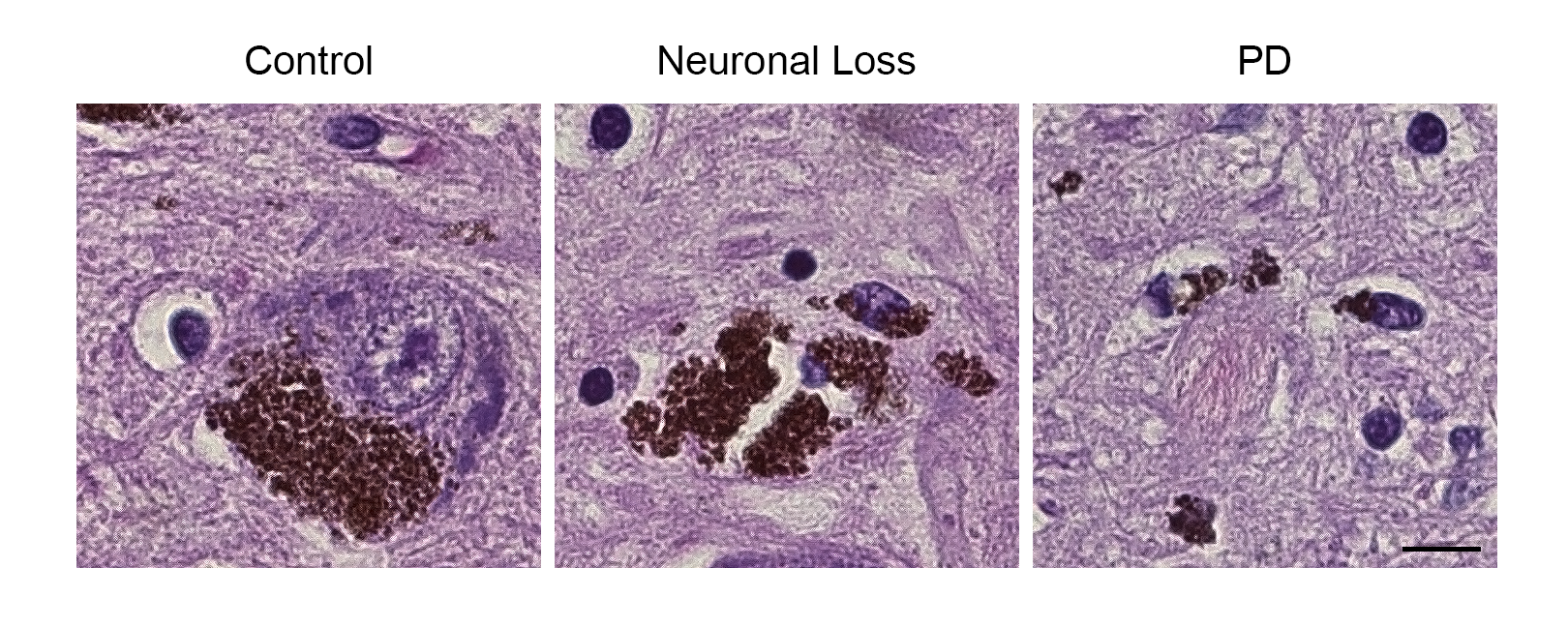
**

Supplemental Figure 7. Presence of pigment-laden macrophages within the brains of controls, patients with evidence of neuronal loss, and confirmed Parkinson’s patients. Tissues were sectioned and stained with H&E and viewed using brightfield microscopy. Scale bar in the 60x field represents 10 μm.


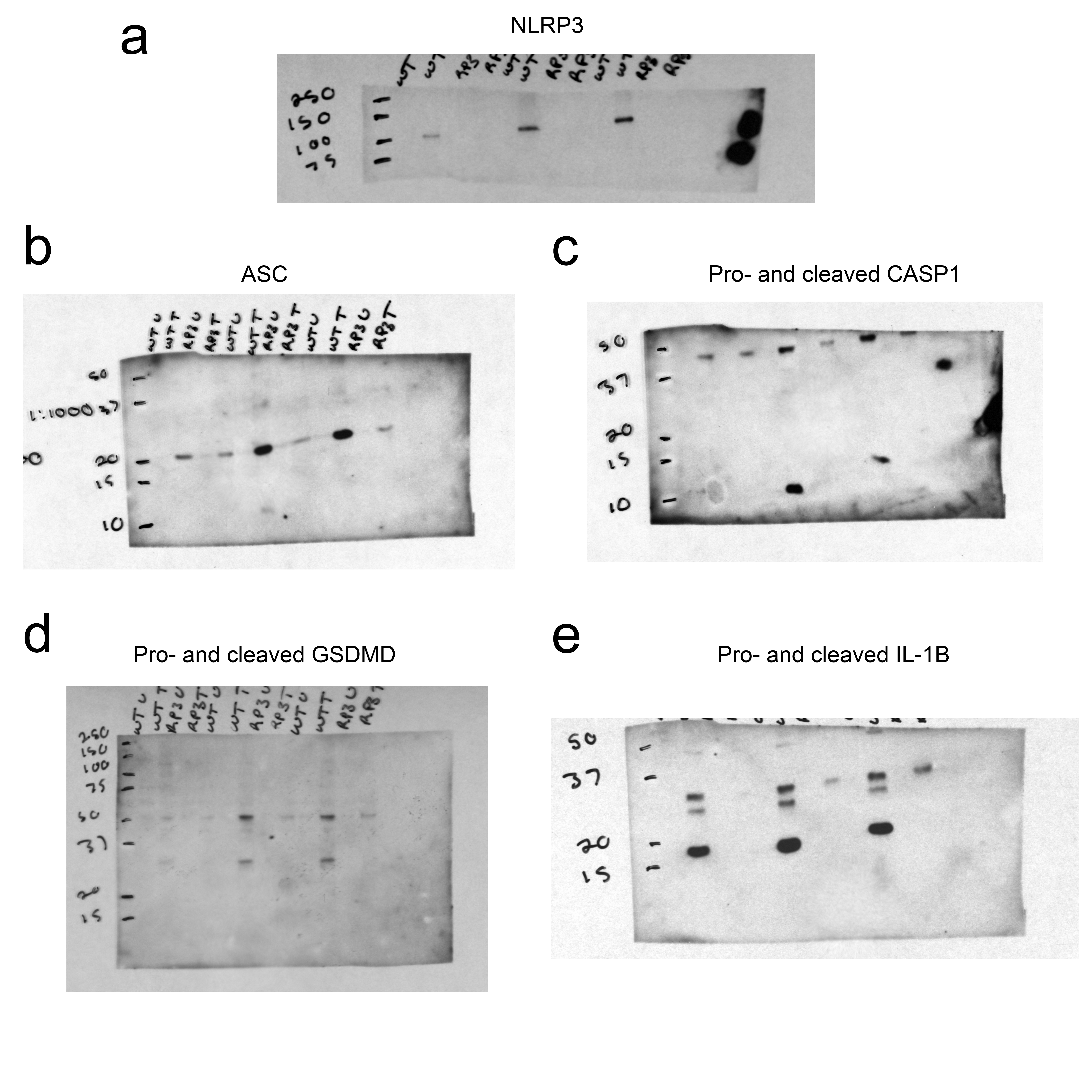


Supplemental Figure 8. Full, uncropped western blots of inflammasome-related proteins in mixed glial cultures from Supplemental Figure 2. Three independent, biologic replicates of mixed glial cultures from WT and *Nlrp3^-/-^* mice were treated with 1 μg/mL LPS for 6 hours followed by 10 μM nigericin-treatment for 30 minutes. Total protein was methanol-precipitated from conditioned media and analyzed using SDS-PAGE for inflammasome-related proteins of interest (see *Methods* for antibodies used). Untreated and treated samples were run in parallel across the three biologic replicates.


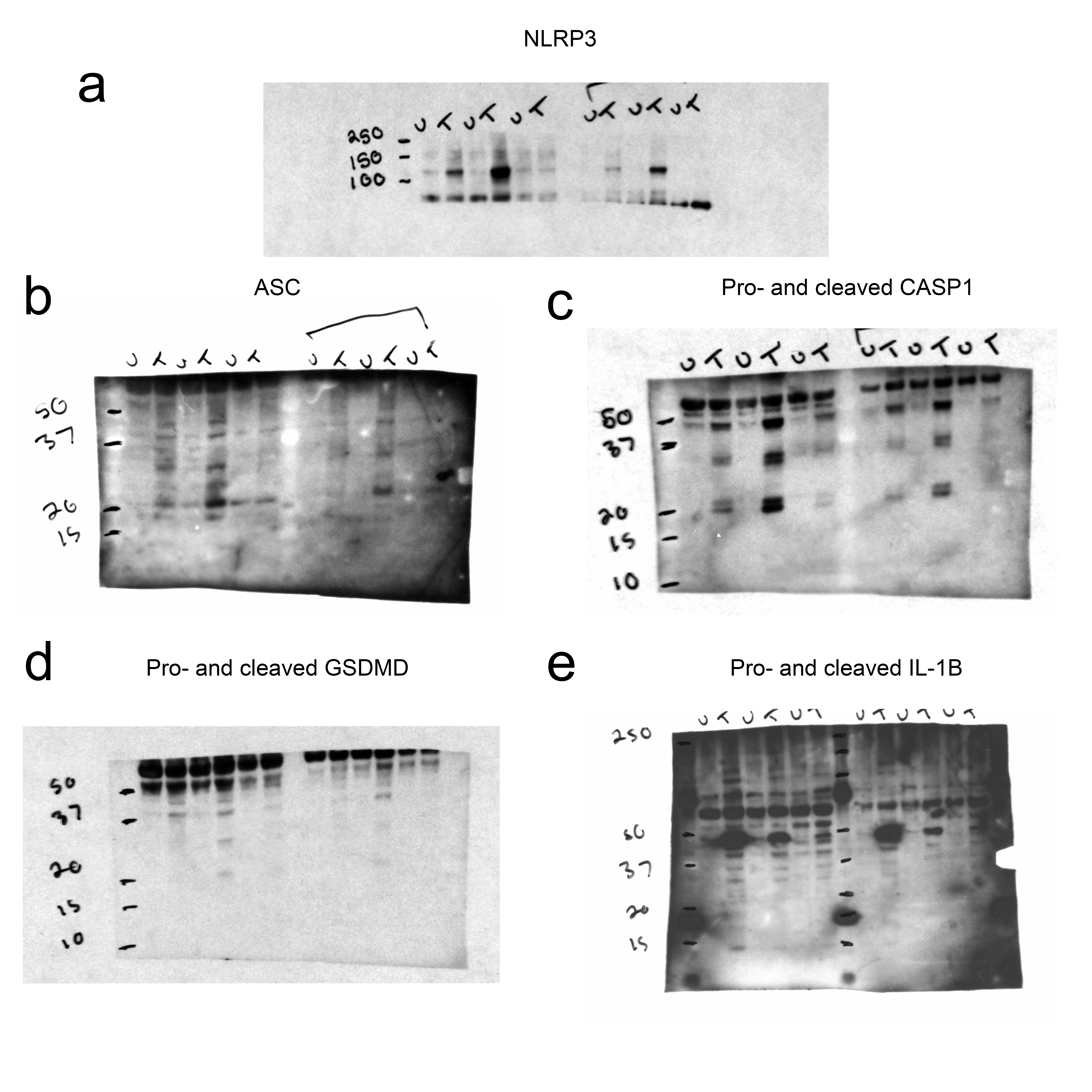


Supplemental Figure 9. Full, uncropped western blots of inflammasome-related proteins in THP-1 cells from Supplemental Figure 3. Three independent, biologic replicates of THP-1 monocytes were treated with 1 μg/mL LPS for 6 hours followed by 10 μM nigericin-treatment for 30 minutes. Total protein was methanol-precipitated from conditioned media and analyzed using SDS-PAGE for inflammasome-related proteins of interest (see *Methods* for antibodies used). Untreated and treated samples were run in parallel across the three biologic replicates. The same samples were diluted 1:2 and run side-by-side for quantification purposes.


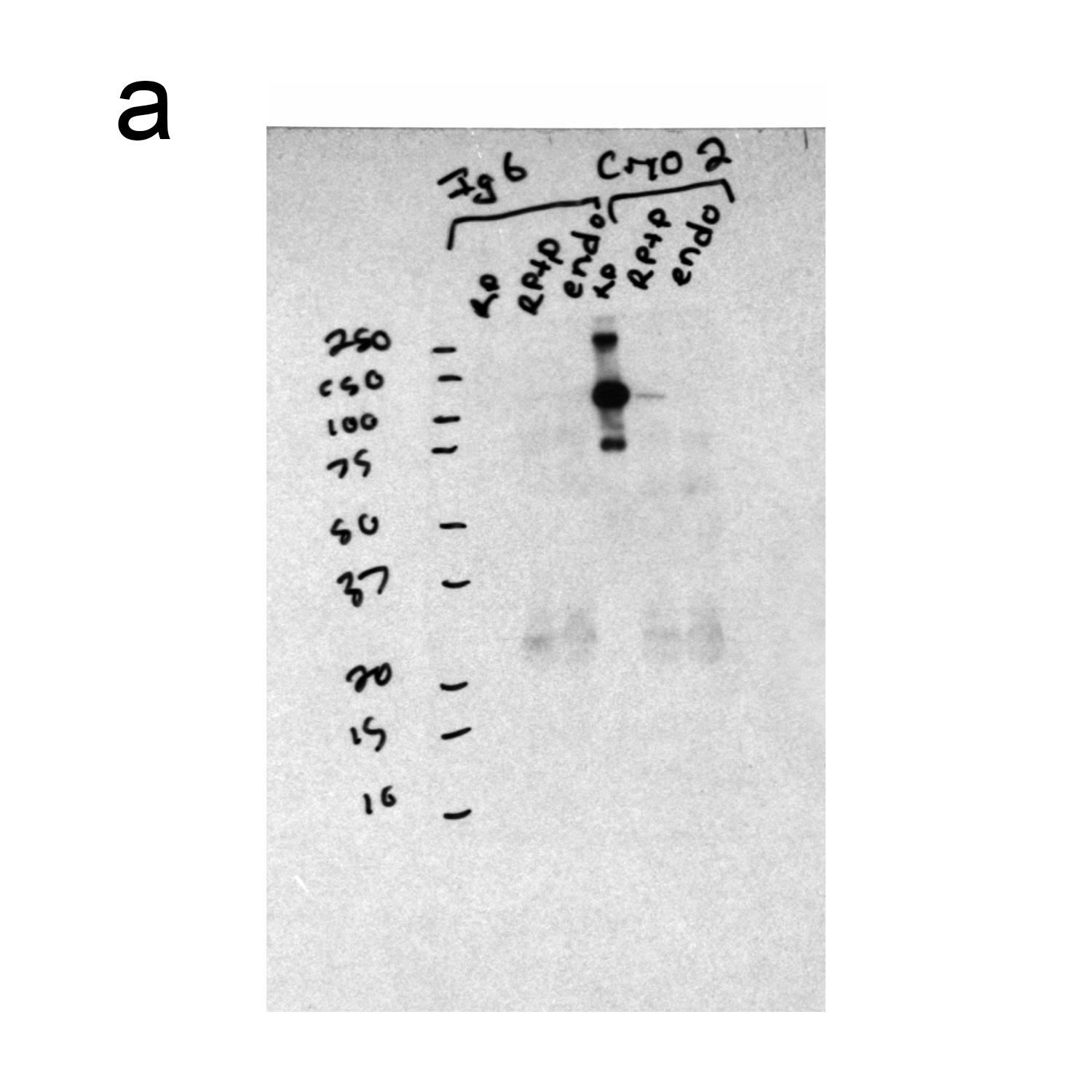


Supplemental Figure 10. Full, uncropped western blot of immunoprecipitation using NLRP3 antibody pair from MSD assay. NLRP3 recombinant protein (RP) was reliably detected in samples of NLRP3 RP in lysis buffer and NLRP3 RP spiked into plasma through immunoprecipitation, where the capture antibody was used as the pull-down antibody and the detection antibody was used as the immunoblotting antibody. The IgG control confirmed antibody pair specificity, as the species-specific IgG was unable to pull-down any NLRP3 RP from these samples.


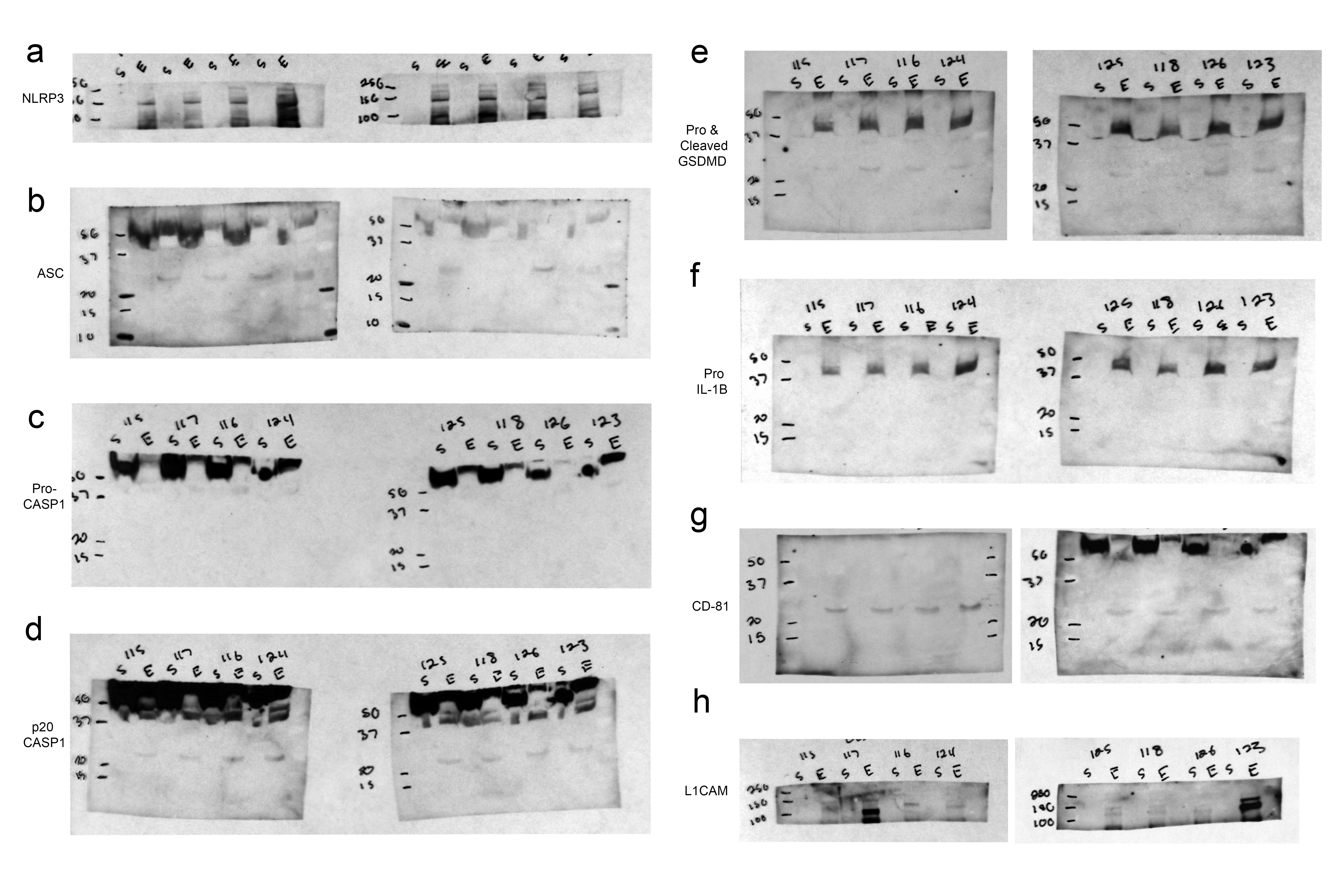


Supplemental Figure 11. Full, uncropped western blots of inflammasome proteins contained within extracellular vesicles isolated from human plasma. Extracellular vesicles (EVs) were isolated from 8 freshly drawn human plasma samples (see *Methods).* The soluble (EV-depleted) and EV fractions were analyzed via SDS-PAGE followed by immunoblotting for inflammasome- and EV-related proteins of interest (see *Methods* for antibodies used).
