# Supplementary material for: Plasma-borne indicators of inflammasome activity in Parkinson’s disease patients
Source: NPJ Parkinsons Dis. 2021 Jan 4;7:2. doi: 10.1038/s41531-020-00147-6 (PMC7782812; doi:10.1038/s41531-020-00147-6)
Supplement: Supplementary file 2 — Reporting Summary Checklist [file 41531_2020_147_MOESM2_ESM.pdf]

nature research

Corresponding author(s): Faith L. Anderson, Katharine M. von Herrmann, Angeline S. Andrew, Yuliya I. Kuras, Alison L. Young, Clemens R. Scherzer, William F. Hickey, Stephen L. Lee, Matthew C. Havrda

Last updated by author(s): 2020/09/09

# Reporting Summary

Nature Research wishes to improve the reproducibility of the work that we publish. This form provides structure for consistency and transparency in reporting. For further information on Nature Research policies, see our Editorial Policies and the Editorial Policy Checklist.

## Statistics

For all statistical analyses, confirm that the following items are present in the figure legend, table legend, main text, or Methods section.

| n/a | Confirmed | |
|---|---|---|
| ☐ | ☒ | The exact sample size ($n$) for each experimental group/condition, given as a discrete number and unit of measurement |
| ☐ | ☒ | A statement on whether measurements were taken from distinct samples or whether the same sample was measured repeatedly |
| ☐ | ☒ | The statistical test(s) used AND whether they are one- or two-sided<br>*Only common tests should be described solely by name; describe more complex techniques in the Methods section.* |
| ☐ | ☒ | A description of all covariates tested |
| ☐ | ☒ | A description of any assumptions or corrections, such as tests of normality and adjustment for multiple comparisons |
| ☐ | ☒ | A full description of the statistical parameters including central tendency (e.g. means) or other basic estimates (e.g. regression coefficient) AND variation (e.g. standard deviation) or associated estimates of uncertainty (e.g. confidence intervals) |
| ☒ | ☐ | For null hypothesis testing, the test statistic (e.g. $F$, $t$, $r$) with confidence intervals, effect sizes, degrees of freedom and $P$ value noted<br>*Give P values as exact values whenever suitable.* |
| ☒ | ☐ | For Bayesian analysis, information on the choice of priors and Markov chain Monte Carlo settings |
| ☒ | ☐ | For hierarchical and complex designs, identification of the appropriate level for tests and full reporting of outcomes |
| ☒ | ☐ | Estimates of effect sizes (e.g. Cohen's $d$, Pearson's $r$), indicating how they were calculated |

*Our web collection on statistics for biologists contains articles on many of the points above.*

## Software and code

Policy information about availability of computer code

| | |
|---|---|
| Data collection | REDCap |
| Data analysis | R, Prism |

For manuscripts utilizing custom algorithms or software that are central to the research but not yet described in published literature, software must be made available to editors and reviewers. We strongly encourage code deposition in a community repository (e.g. GitHub). See the Nature Research guidelines for submitting code & software for further information.

## Data

Policy information about availability of data

All manuscripts must include a data availability statement. This statement should provide the following information, where applicable:
- Accession codes, unique identifiers, or web links for publicly available datasets
- A list of figures that have associated raw data
- A description of any restrictions on data availability

In report: Mouse genomic data are publicly available at the Broad Single Cell Portal (https://portals.broadinstitute.org/single_cell), raw dataset. GEO: (https://www.ncbi.nlm.nih.gov/geo/query/acc.cgi?acc=GSE129788), original publication [51].

# Field-specific reporting

Please select the one below that is the best fit for your research. If you are not sure, read the appropriate sections before making your selection.

☒ Life sciences ☐ Behavioural & social sciences ☐ Ecological, evolutionary & environmental sciences

For a reference copy of the document with all sections, see nature.com/documents/nr-reporting-summary-flat.pdf

# Life sciences study design

All studies must disclose on these points even when the disclosure is negative.

| | |
|---|---|
| Sample size | Based on patient recruiting. |
| Data exclusions | Exclusion criteria: Pregnant women, drug-induced parkinsonism, acute or chronic infection, traumatic injury within the last 90 days, exposure to corticosteroids within the last 90 days, neoplastic disease (cancer). |
| Replication | Studies were reported from analysis of a combined dataset. Similar trends were apparent, however, sample number was not sufficient in either dataset alone to achieve statistical significance. |
| Randomization | N/A |
| Blinding | Non-clinical experimenters were blinded to patient identity and group throughout the development and execution of immunosorbent assays. |

# Reporting for specific materials, systems and methods

We require information from authors about some types of materials, experimental systems and methods used in many studies. Here, indicate whether each material, system or method listed is relevant to your study. If you are not sure if a list item applies to your research, read the appropriate section before selecting a response.

## Materials & experimental systems

| n/a | Involved in the study | |
|---|---|---|
| ☐ | ☒ | Antibodies |
| ☐ | ☒ | Eukaryotic cell lines |
| ☒ | ☐ | Palaeontology and archaeology |
| ☐ | ☒ | Animals and other organisms |
| ☐ | ☒ | Human research participants |
| ☒ | ☐ | Clinical data |
| ☒ | ☐ | Dual use research of concern |

## Methods

| n/a | Involved in the study | |
|---|---|---|
| ☒ | ☐ | ChIP-seq |
| ☒ | ☐ | Flow cytometry |
| ☒ | ☐ | MRI-based neuroimaging |

## Antibodies

| | |
|---|---|
| Antibodies used | In report: The following antibodies were used for the detection of inflammasome-related proteins in cell culture supernatant: anti-NLRP3 (AG-20B-0014, Adipogen, San Diego, CA; 15101, Cell Signaling Technology, Danvers, MA), anti-CASP1 (p20) (AG-20B-0048B, Adipogen, San Diego, CA), anti-CASP1 (p10) (AG-20B-0042, Adipogen, San Diego, CA), anti-GSDMD (93709, Cell Signaling Technology, Danvers, MA), anti-ASC (AG-25B-0006, Adipogen, San Diego, CA; 67824, Cell Signaling Technology, Danvers, MA), and anti-IL-1B (NB600-633, Novus Biologicals, Centennial, CO; AF-401, R&D Systems, Minneapolis, MN). The antibody pair: capture, Cryo 2 (AG-20B-0014-C100, Adipogen, San Diego, CA) and detection, D4D8T (15101, Cell Signaling Technologies, Danvers, MA) was used in the immunsorbent assay. For antibody pair validation, immunoprecipitation assays used the same capture and detection antibodies from the electrochemiluminescent assays as the pull-down and blotting antibodies respectively. The antibodies used for the human EV lysates were the same as those above used for the detection of inflammasome-related proteins in THP-1 cell culture supernatant. |
| Validation | Described, using recombinant protein and null cells (see Figure 2, Supplemental Figure 2). |

## Eukaryotic cell lines

Policy information about cell lines

| | |
|---|---|
| Cell line source(s) | Primary glial cultures, THP-1 cells |
| Authentication | Mixed glial cultures from mouse embryos are routinely established in the lab (see "Animals and other organisms") and reported SDS-PAGE experiments confirm robust inflammasome activity in these cultures. THP-1 cells were purchased from ATCC and used within 20 passages. |

| | |
|---|---|
| Mycoplasma contamination | Primary cultures are not maintained for more than 10 days, THP-1 cells are certified mycoplasma free by ATCC and we use at an early passage. All cell line data in the report is provided for verification purposes only and is consistent with cited reports using identical cell systems. Routine screening of cells is performed during ongoing experiments using Hoescht dye and microscopic analysis. |
| Commonly misidentified lines (See ICLAC register) | N/A |

## Animals and other organisms

Policy information about studies involving animals; ARRIVE guidelines recommended for reporting animal research

| | |
|---|---|
| Laboratory animals | Postnatal day 0-3 C57BL/6J WT and Nlrp3-/- mice of mixed sexes were used to establish primary glial cultures. |
| Wild animals | N/A |
| Field-collected samples | N/A |
| Ethics oversight | All mouse experiments were conducted according to the ARRIVE guidelines and approved by the Institutional Animal Care and Use Committee (IACUC) at Dartmouth protocol entitled “Animal Models of Neurodegenerative Diseases” protocol #00002117. |

Note that full information on the approval of the study protocol must also be provided in the manuscript.

## Human research participants

Policy information about studies involving human research participants

| | |
|---|---|
| Population characteristics | See Table 1. Inclusion for PD: A diagnosis of parkinsonism. This can include idiopathic Parkinson’s disease. Over 21 years of age. Controls: No diagnosis of parkinsonism or other neurologic disorder. Over 21 years of age. |
| Recruitment | Recruited in person during routine clinical care. Recruited based on response to press releases. |
| Ethics oversight | Dartmouth-Hitchcock Medical Center, Committee for the Protection of Human Subjects. |

Note that full information on the approval of the study protocol must also be provided in the manuscript.
